# Cost-effectiveness analysis of first-line treatments for recurrent or metastatic head and neck cancer in China: an economic evaluation based on network meta-analysis

**DOI:** 10.3389/fphar.2025.1644426

**Published:** 2025-09-30

**Authors:** Fen Liu, Yuhang Liu, Guilin Song, Yong Pan, Qiao Xia, Haonan Li

**Affiliations:** ^1^ Department of Pharmacy, Hunan Cancer Hospital, The Affiliated Cancer Hospital of Xiangya School of Medicine, Central South University, Changsha, Hunan, China; ^2^ School of Medical Business, Guangdong Pharmaceutical University, Guangzhou, Guangdong, China; ^3^ Department of Chemical Medicine, Yantai Center for Food And Drug Control, Yantai, China; ^4^ School of Pharmaceutical Sciences, Peking University, Beijing, China

**Keywords:** cost-effectiveness, head and neck squamous cell carcinoma, cetuximab, pembrolizumab, finotonlimab

## Abstract

**Background:**

Recurrent or metastatic head and neck squamous cell carcinoma (R/M HNSCC) is a common pathological type of head and neck tumors, imposing a huge disease burden in China. This study evaluated the cost-effectiveness of three first-line treatment regimens for R/M HNSCC approved in China from the perspective of Chinese payers, including cetuximab plus chemotherapy, pembrolizumab as monotherapy or in combination with chemotherapy, and finotonlimab plus chemotherapy, aiming to provide reference for decision-making.

**Methods:**

Based on the data from three randomized controlled trials: KEYNOTE-048 (NCT02358031), CHANGE-2 (NCT02383966), and the finotonlimab trial (NCT04146402), we conducted a network meta-analysis and employed partitioned survival model (PSM) to indirectly evaluate and compare the cost-effectiveness of treatments associated with finotonlimab, pembrolizumab (monotherapy or combination), and cetuximab. The simulation cycle of the model was set to 3 weeks, with a study duration of 20 years and a discount rate of 3.0%. The primary outcomes included life years (LYs), quality-adjusted life years (QALYs), incremental cost-effectiveness ratios (ICERs) and incremental net monetary benefits (INMBs), with a willingness-to-pay (WTP) threshold of 1–3 times China’s *per capita* gross domestic product (GDP). Furthermore, subgroup analyses, sensitivity analyses, and scenario analyses were performed to validate the robustness of the findings.

**Results:**

In the overall population, compared to cetuximab-chemotherapy, pembrolizumab monotherapy (ICER: 85,131.70/QALY) and pembrolizumab-chemotherapy (ICER: 203,545.22/QALY) were less cost-effective, while finotonlimab-chemotherapy (ICER: 161.13/QALY) was significantly more favorable. The net monetary benefit (NMB) analysis supported this finding, with finotonlimab-chemotherapy group having the highest INMB ($4,746.03 vs cetuximab-chemotherapy), followed by pembrolizumab (-$17,381.75) and pembrolizumab-chemotherapy (-$32,841.18). The results were similar in the population with PD-L1 CPS ≥1 and CPS ≥20. The one-way sensitivity analysis revealed that drug costs, the discount rate, and utility values for progression-free survival (PFS) and disease progression (PD) were key parameters significantly impacting the ICERs. Additionally, both probabilistic sensitivity analysis and scenario analysis confirmed that the results of base-case analysis were robust.

**Conclusion:**

From the perspective of the Chinese population, finotonlimab-chemotherapy is the most cost-effective first-line treatment for R/M HNSCC, followed by cetuximab-chemotherapy. Pembrolizumab, whether as monotherapy or in combination, does not offer economic benefits.

## 1 Introduction

Head and neck squamous cell carcinoma (HNSCC) represents the most prevalent pathological subtype, accounting for approximately 90% of all head and neck tumors (Bray et al., 2024). Notably, Asia, especially Southeast Asia (including countries such as China and South Korea as well as other regions), is recognized as a high-incidence area for HNSCC. According to the statistics of the National Cancer Center in 2024, China reported approximately 94,600 new cases and 52,100 deaths annually due to HNSCC, excluding nasopharyngeal carcinoma. The incidence rate of HNSCC ranked sixth among all systemic malignant tumors, while its mortality rate ranked seventh (Han et al., 2024). HNSCC demonstrates substantial heterogeneity and invasiveness. Approximately 60% of patients are diagnosed at a locally advanced stage at the time of initial presentation. Despite the implementation of comprehensive treatment modalities, such as surgery, radiotherapy, and/or chemotherapy, 40%–60% of patients still experience local recurrence or distant metastasis following treatment (Han et al., 2024).

Since the 1990s, platinum-based chemotherapy has been the standard first-line treatment for recurrent/metastatic (R/M) HNSCC (Forastiere et al., 1992; Gibson et al., 2005). However, their effect on improving patient survival has remained limited. With the advancement of targeted therapies, the combination of cetuximab and platinum-based chemotherapy has demonstrated significant progress in R/M HNSCC, increasing the median overall survival (OS) to 10.1 months and decreasing the mortality risk by 20% compared to the chemotherapy-only group (Vermorken et al., 2008). In recent years, the success of the KEYNOTE-048 study has marked the beginning of the immunotherapy era for R/M HNSCC (Burtness et al., 2019). Pembrolizumab monotherapy respectively prolonged the OS to 14.9 months and 12.3 months in patients with programmed cell death one ligand-1(PD-L1) combined positive score (CPS) ≥ 20 and CPS ≥1, resulting in a 39% and 22% reduction in the risk of mortality compared with the cetuximab plus chemotherapy group. However, no significant advantage was observed for pembrolizumab monotherapy in the total population. In contrast, pembrolizumab plus chemotherapy, compared with cetuximab plus chemotherapy group, demonstrated a more pronounced benefit, reducing the risk of death by 38%, 36%, and 29% in patients with CPS ≥20, CPS ≥1, and the total population, respectively. Based on the positive findings, the combination of cetuximab and chemotherapy, pembrolizumab monotherapy (CPS ≥1), or combined with chemotherapy, have been recommended as first-line treatment regime in the 2025 Chinese Society of Clinical Oncology (CSCO) Guidelines. The latest randomized controlled trial (RCT) (Shi et al., 2024) demonstrated that the combination of finotonlimab with chemotherapy significantly prolonged OS compared to platinum-based chemotherapy alone (14.1 months vs. 10.5 months, hazard ratio [HR] = 0.73, 95% confidence interval [CI]: 0.57–0.95, P = 0.0165). This research positive outcome directly resulted in the approval by the National Medical Products Administration (NMPA) for the indication of finotonlimab in combination with platinum-based chemotherapy as a first-line treatment for R/M HNSCC in 2025. In the same year, this therapeutic regimen was officially incorporated into the CSCO guidelines. Notably, finotonlimab marks the first domestically developed PD-1 monoclonal antibody to receive approval for the treatment of R/M HNSCC in China.

Cetuximab or immune checkpoint inhibitors (ICIs)significantly prolonged the OS of patients with R/M HNSCC compared to the chemotherapy-only group. These therapies also resulted in a heavier cost burden for patients. Current economic evaluations predominantly focus on comparing the cost-effectiveness of pembrolizumab as monotherapy or in combination with chemotherapy *versus* cetuximab plus chemotherapy. In contrast, there remains a relative paucity of economic evaluations comparing finotonlimab with other established first-line treatment options. In randomized controlled trials (RCTs) conducted for the approval of new drugs, the control group typically receives standard therapy or placebo. However, when assessing the efficacy of new drugs in comparison to existing treatment regimens, direct comparative data are often limited or unavailable. In such cases, indirect comparison methods or network meta-analysis (NMA) can serve as valuable tools for evaluating the relative effectiveness of multiple interventions across different patient populations (Guo, 2022). NMA is a statistical methodology that extends beyond traditional pairwise comparisons, enabling the simultaneous evaluation and ranking of multiple treatment strategies in terms of overall efficacy. This approach is increasingly utilized in pharmacoeconomic evaluations (Dzienis et al., 2025; Xiang et al., 2024; Zhao et al., 2022). Therefore, from the perspective of Chinese healthcare payers, this study employs an indirect comparison method to evaluate and compare the cost-effectiveness of finotonlimab, pembrolizumab, and cetuximab in the first-line treatment of R/M HNSCC.

## 2 Materials and methods

### 2.1 Model construction

This study employed a partitioned survival model (PSM) to evaluate the costs and effectiveness of finotonlimab plus chemotherapy (finotonlimab-chemo), pembrolizumab, pembrolizumab plus chemotherapy (pembrolizumab-chemo), or cetuximab plus chemotherapy (cetuximab-chemo) as first-line treatments for R/M HNSCC, from the perspective of Chinese payers. The model cycle length was set at 3 weeks, with a time horizon of 20 years, capturing 99.5% of mortality events across all groups, effectively simulating the lifetime course of HNSCC patients. Both costs and outcomes were discounted at an annual rate of 3.0% (Goldstein et al., 2015). Primary outcome measures included total costs, life years (LYs), quality-adjusted life years (QALYs), incremental cost-effectiveness ratios (ICERs), net monetary benefits (NMB), and incremental net monetary benefits (INMB). The willingness-to-pay (WTP) threshold was set at 1 to 3 times China’s *per capita* GDP in 2023, equivalent to $12,296.7-$36,890.1 (Based on national statistical data). The PSM was developed using Microsoft Excel 2019.

**FIGURE 1:**
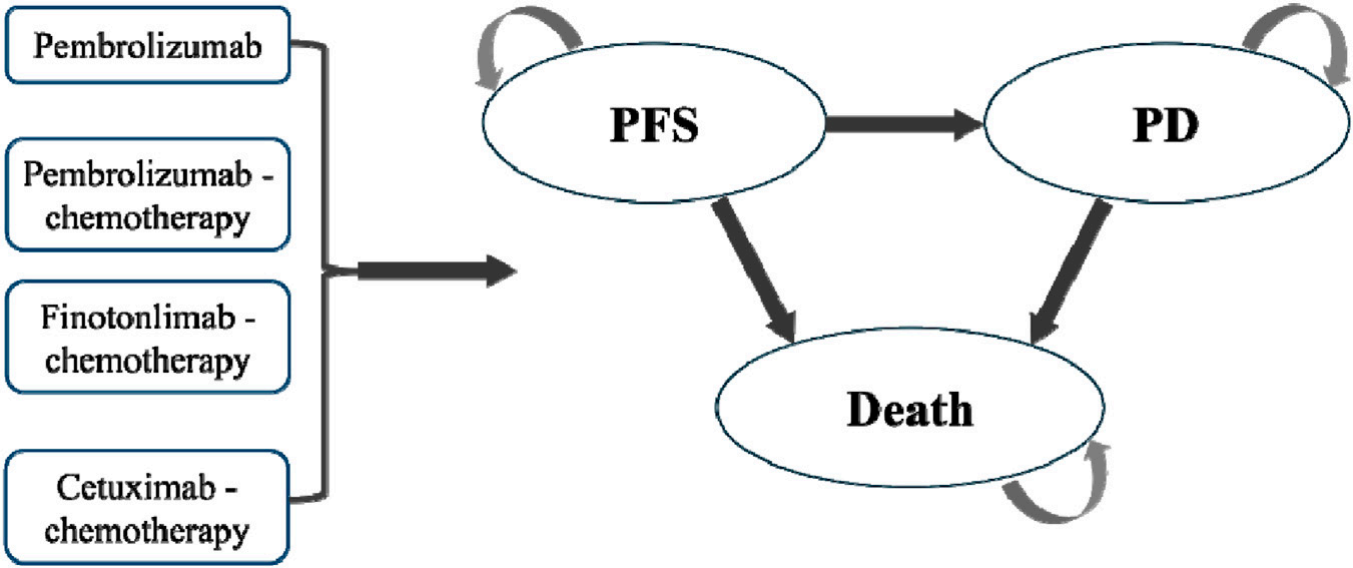
Partitioned survival model structure.

The model structure comprised three health states for R/M HNSCC: progression-free survival (PFS), progressive disease (PD), and death (Figure 1). All patients initially received first-line therapy (finotonlimab-chemo, pembrolizumab, pembrolizumab-chemo, or cetuximab-chemo) until disease progression or unacceptable toxicity (Figure 1). Upon progression, patients transitioned to second-line therapy (primarily taxane-based chemotherapy or best supportive care). In the base-case analysis, all patients were assumed to receive BSC for 3 months before death.

### 2.2 Clinical data

This study utilized data from the reported results of KEYNOTE-048 (Burtness et al., 2019), CHANGE-2 (Guo et al., 2021), and the finotonlimab clinical trial (Shi et al., 2024). As shown in Table 1, the baseline characteristics of patients across the three trials-including age, sex ratio, disease status, and primary tumor location-demonstrated good consistency. For bias risk assessment, we employed RevMan 5.4 software and selected a fixed-effects model due to the limited data available for evaluating inter-trial heterogeneity. The overall methodological quality of the included studies was high, though one study exhibited a selection bias risk due to inadequate allocation concealment, and two studies showed performance bias risks related to blinding issues (Supplementary Figure S1). In the statistical analysis phase, we performed a Bayesian network meta-analysis using the ‘meta’ and ‘netmeta’ packages in R version 4.3.1 (Supplementary Figure S2). This approach allowed us to derive hazard ratios (HRs) for OS and PFS across different treatment regimens, including finotonlimab plus chemotherapy, pembrolizumab monotherapy, pembrolizumab plus chemotherapy, and cetuximab plus chemotherapy. Finally, the pooled HRs were applied in the subsequent cost-effectiveness analysis (Supplementary Figures S3, S4).

**TABLE 1:** Baseline characteristics of the clinical research.

Group	KEYNOTE-048		
	Pembrolizumab	Pembrolizumab-chemo	Cetuximab-chemo
Number	301	281	300
Age (mean and range)	62.0 (56.0–68.0)	61.0 (55.0–68.0)	61.0 (54.5–68.0)
Male (proportion of males, %)	83% (250/301)	80% (224/281)	87% (261/300)
PD-L1 CPS (proportion ≥1, %)	257 (85%)	255 (85%)	242 (86%)
Disease status (proportion of Metastatic, %)	216 (72%)	201 (72%)	203 (68%)
Primary tumor location (Hypopharynx, %)	38 (13%)	44 (16%)	39 (13%)

Group	Finotonlimab trial		CHANGE-2	
	Finotonlimab-chemo	Chemo	Cetuximab-chemo	Chemo
Number	301	123	164	79
Age (mean and range)	62.0 (56.0–68.0)	60.0 (32.0–77.0)	57.1 (28.0–82.0)	57.0 (34.0–77.0)
Male (proportion of males, %)	83% (250/301)	89.4% (110/123)	89.0% (146/164)	84.8% (67/79)
PD-L1 CPS (proportion ≥1, %)	257 (85%)	123 (94%)	-	-
Disease status (proportion of Metastatic, %)	216 (72%)	87 (71%)	91 (55.5%)	38 (48.1%)
Primary tumor location (Hypopharynx, %)	38 (13%)	29 (23.6%)	42 (25.6%)	19 (24.1%)

### 2.3 Model survival and progression estimates

Survival data from PFS and OS curves were extracted using GetData Graph Digitizer (v2.26). Since individual patient-level data were unavailable, we reconstructed time-to-event data using Guyot’s algorithm (Guyot et al., 2012), a validated approach for accurately estimating survival outcomes from published Kaplan-Meier curves. To model expected survival for different treatment arms (pembrolizumab, pembrolizumab-chemotherapy, finotonlimab-chemotherapy, and cetuximab-chemotherapy), we applied the respective hazard ratios (HRs) to the reference survival curve. The reference chemotherapy PFS and OS curves were obtained from the CHANGE-2 trial (Guo et al., 2021), selected due to their high data maturity (>75% for OS and >60% for PFS). We fitted the extracted survival data to multiple parametric distributions (weibull, log-normal, log-logistic, exponential, gamma, generalized gamma, and gompertz) and determined the optimal model based on the goodness-of-fit was visually assessed by examining the alignment between modeled and observed survival curves (Table 2; Supplementary Figures S5-S6). Model validity was confirmed through residual analysis (Supplementary Tables S1, S2). This multi-criteria approach ensured the most appropriate survival function was chosen for subsequent analyses.

**TABLE 2:** Model parameters, baseline values, ranges, and distributions for sensitivity analyses.

Parameter	Baseline value	Minimum	Maximum	Distribution	Reference
Survival model					
Lognormal OS survival model of chemotherapy	meanlog: 2.062 sdlog: 0.903	-	-	-	Model fitting
Lognormal PFS survival model of chemotherapy	meanlog: 1.316 sdlog: 0.745	-	-	-	Model fitting
HR for OS (cetuximab-chemo vs chemotherapy)	0.85	0.62	1.16	Lognormal	Network meta-analysis
HR for PFS (cetuximab-chemotherapy vs chemotherapy)	0.78	0.55	1.11	Lognormal	Network meta-analysis
HR for OS (finotonlimab plus chemotherapy vs chemotherapy)	0.87	0.68	1.13	Lognormal	Network meta-analysis
HR for PFS (finotonlimab plus chemotherapy vs chemotherapy)	0.89	0.69	1.16	Lognormal	Network meta-analysis
HR for OS (pembrolizumab vs chemotherapy)	0.78	0.55	1.12	Lognormal	Network meta-analysis
HR for PFS (pembrolizumab vs chemotherapy)	0.89	0.60	1.31	Lognormal	Network meta-analysis
HR for OS (pembrolizumab plus chemotherapy vs chemotherapy)	0.76	0.53	1.10	Lognormal	Network meta-analysis
HR for PFS (pembrolizumab plus chemotherapy vs chemotherapy)	0.76	0.51	1.12	Lognormal	Network meta-analysis
Cost of drug, $					
Finotonlimab (100 mg)	674.00	539.20	808.80	Gamma	Market consultation
Pembrolizumab (100 mg)	2,515.97	2,012.78	3,019.17	Gamma	Www.drugs.com
Cetuximab (100 mg)	150.53	120.42	180.64	Gamma	Www.drugs.com
Cisplatin (10 mg)	1.12	0.90	1.34	Gamma	Www.drugs.com
5-Fluorouracil (250 mg)	7.65	6.12	9.18	Gamma	Www.drugs.com
Taxane (60 mg)	16.23	12.98	19.48	Gamma	Www.drugs.com
Cost pert cycle. $					
Laboratory	97.51	78.00	117.01	Gamma	Pei et al. (2023)
Tumor imaging	209.08	167.27	250.90	Gamma	Pei et al. (2023)
Administration	48.25	38.60	57.90	Gamma	Pei et al. (2023)
Best supportive care	142.74	114.19	171.29	Gamma	Pei et al. (2023)
Terminal care per patient	1,842.55	1,474.04	2,211.06	Gamma	Pei et al. (2023)
Cost of serious adverse events pert cycle. $					
Anemia	44.91	35.93	53.90	Gamma	Xiang et al. (2024)
Febrile neutropenia	295.83	236.67	355.00	Gamma	Xiang et al. (2024)
Leukopenia	119.98	95.99	143.98	Gamma	Xiang et al. (2024)
Neutropenia	119.98	95.99	143.98	Gamma	Xiang et al. (2024)
Thrombocytopenia	400.74	320.59	480.89	Gamma	Xiang et al. (2024)
Nausea	17.71	14.17	21.25	Gamma	Xu et al. (2024b)
Stomatitis	57.42	45.93	68.90	Gamma	Xu et al. (2024b)
Fatigue	89.51	71.61	107.41	Gamma	Xiang et al. (2024)
Mucosal inflammation	57.42	45.93	68.90	Gamma	Xu et al. (2024b)
Pneumonia	629.90	503.92	755.88	Gamma	Xu et al. (2023)
Neutrophil count decreased	119.98	95.99	143.98	Gamma	Xiang et al. (2024)
Platelet count decreased	400.74	320.59	480.89	Gamma	Xiang et al. (2024)
White blood cell count decreased	119.98	95.99	143.98	Gamma	Xiang et al. (2024)
Hypokalaemia	14.04	11.23	16.85	Gamma	Local charge
Hyponatraemia	14.04	11.23	16.85	Gamma	Local charge
Rash	1.86	1.49	2.23	Gamma	Wang et al. (2020)
Utility					
PFS	0.82	0.66	0.98	Beta	Borse et al. (2022)
PD	0.78	0.62	0.94	Beta	Borse et al. (2022)
Disutilities for adverse events	−0.02	−0.015	−0.025	Beta	Borse et al. (2022)
Discount rate (%)	3	2.5	3.5	Beta	Goldstein et al. (2015)
Body surface area (m^2^)	1.65				Shi et al. (2024)
Body weight (kg)	59				Shi et al. (2024)
Risk of serious adverse events(%)					
Cetuximab-Chemotherapy group					
Anaemia	17.07	-	-	Fixed	Guo et al. (2021)
Febrile neutropenia	5.92	-	-	Fixed	Guo et al. (2021)
Leukopenia	5.57	-	-	Fixed	Guo et al. (2021)
Neutropenia	21.25	-	-	Fixed	Guo et al. (2021)
Thrombocytopenia	9.06	-	-	Fixed	Guo et al. (2021)
Nausea	5.92	-	-	Fixed	Guo et al. (2021)
Fatigue	4.88	-	-	Fixed	Guo et al. (2021)
Mucosal inflammation	5.23	-	-	Fixed	Guo et al. (2021)
Pneumonia	6.97	-	-	Fixed	Guo et al. (2021)
Neutrophil count decreased	12.89	-	-	Fixed	Guo et al. (2021)
White blood cell count decreased	9.06	-	-	Fixed	Guo et al. (2021)
Hypokalaemia	5.92	-	-	Fixed	Guo et al. (2021)
Hyponatraemia	5.92	-	-	Fixed	Guo et al. (2021)
Rash	5.92	-	-	Fixed	Guo et al. (2021)
Pembrolizumab group					
Pneumonia	5.67	-	-	Fixed	Burtness et al. (2019)
Hyponatraemia	6.00	-	-	Fixed	Burtness et al. (2019)
Pembrolizumab-chemotherapy group					
Anaemia	25.36	-	-	Fixed	Burtness et al. (2019)
Febrile neutropenia	8.70	-	-	Fixed	Burtness et al. (2019)
Neutropenia	17.75	-	-	Fixed	Burtness et al. (2019)
Thrombocytopenia	9.06	-	-	Fixed	Burtness et al. (2019)
Nausea	5.80	-	-	Fixed	Burtness et al. (2019)
Stomatitis	8.33	-	-	Fixed	Burtness et al. (2019)
Fatigue	7.25	-	-	Fixed	Burtness et al. (2019)
Mucosal inflammation	9.78	-	-	Fixed	Burtness et al. (2019)
Pneumonia	5.43	-	-	Fixed	Burtness et al. (2019)
Neutrophil count decreased	10.87	-	-	Fixed	Burtness et al. (2019)
Platelet count decreased	5.43	-	-	Fixed	Burtness et al. (2019)
White blood cell count decreased	5.43	-	-	Fixed	Burtness et al. (2019)
Hypokalaemia	6.52	-	-	Fixed	Burtness et al. (2019)
Hyponatraemia	7.97	-	-	Fixed	Burtness et al. (2019)
Finotonlimab-chemotherapy group					
Anemia	8.10	-	-	Fixed	Shi et al. (2024)

### 2.4 Model survival and transition probabilities

From the viewpoint of healthcare payers in China, the economic evaluation accounted for direct medical costs, such as costs for drug procurement, laboratory tests, tumor imaging, treatment administration, best supportive care, end-of-life care, and management of adverse events (see Table 2). The treatment regimen and dosing schedule were detailed in Supplementary Table S3. Drug costs were calculated based on body surface area (1.65 m^2^) and body weight (59 kg) (Shi et al., 2024), with unit prices sourced from gamma-distributed 2023 market data: finotonlimab, pembrolizumab, and cetuximab, conventional chemotherapy agents included cisplatin, 5-fluorouracil, and taxane (Table 2).

Procedure costs per cycle were derived from published Chinese studies: laboratory tests, tumor imaging, drug administration, best supportive care, and terminal care. Adverse event costs included serious adverse events (Grade ≥3) with an incidence rate of 5% or higher, such as hematologic toxicities (febrile neutropenia and thrombocytopenia) and non-hematologic events (pneumonia and stomatitis). All values were adjusted to 2023 USD (exchange rate: 1 USD = 7.08 CNY).

Health state utilities were modeled using beta distributions: PFS utility 0.82 and PD utility 0.78, derived from Chinese-specific HNSCC studies. Treatment-related adverse events incurred a uniform disutility of −0.02 per episode and a 3% annual discount rate was applied to both costs and outcomes with detailed parameters and distributions provided in Table 2.

### 2.5 Sensitivity analysis and scenario analysis

This study employed both one-way sensitivity analysis and probabilistic sensitivity analysis to evaluate model robustness. The one-way sensitivity analysis identified key parameters significantly impacting ICER values, allowing comprehensive assessment of uncertainty from all model parameters (Heppner et al., 2022), with results visualized via tornado diagrams.

We conducted probabilistic sensitivity analysis to comprehensively evaluate parameter uncertainty by assigning specific probability distributions to each parameter. Cost parameters were modeled using gamma distributions, while health utility parameters followed beta distributions. Through 1,000 Monte Carlo simulations, we generated cost-effectiveness scatterplots and acceptability curves. These visualizations demonstrate the probability of each treatment regimen (pembrolizumab monotherapy, pembrolizumab plus chemotherapy, finotonlimab plus chemotherapy, and cetuximab plus chemotherapy) being cost-effective across a range of willingness-to-pay thresholds. The complete specifications of parameter ranges and their corresponding distribution types are provided in Table 2.

To evaluate the robustness of our cost-effectiveness findings, we conducted four scenario analyses: (1) We assessed the impact of drug price fluctuations by modeling ±20% variations in the costs of finotonlimab, pembrolizumab, and cetuximab, reflecting potential market changes or reimbursement adjustments. (2) We extended the treatment duration for pembrolizumab by 3 months to account for real-world clinical scenarios where patients may continue therapy beyond standard protocols due to delayed progression assessments or perceived clinical benefit, which was adopted by similar studies (Zhao et al., 2022; Su et al., 2021). (3) We simulated the impact of anticipated future price reductions for key drugs based on foreseeable market events within the Chinese healthcare landscape. For finotonlimab, we assumed its entry into the National Reimbursement Drug List (NRDL) in 2025, corresponding to year two of our model, followed by a price reduction of 60% thereafter-consistent with the typical discount achieved during China’s national medical insurance negotiations. For pembrolizumab, we projected a patent expiration and subsequent introduction of biosimilars into the Chinese market around 2028 (year four of the model), which would trigger a comparable 60% price reduction. Although biosimilars of cetuximab are already available, we applied a uniform 60% price reduction starting in 2028 to reflect potential intensified competition specific to the head and neck cancer indication and to ensure consistency across comparative treatment arms. (4) To evaluate the impact of the model’s time horizon on long-term outcomes, we also extended the simulation period to 30 years. These scenarios were selected to test the model’s sensitivity to critical economic and treatment duration parameters that could significantly influence the cost-effectiveness conclusions.

### 2.6 Subgroup analysis

In the subgroup analysis, the ICER was calculated using the subgroup-specific HRs for PFS and OS obtained from the KEYNOTE-048 (Burtness et al., 2019), CHANGE-2 (Guo et al., 2021), and the finotonlimab trial (Shi et al., 2024) studies. In the China scenario, subgroup analyses were conducted under WTP thresholds of 1–3 times the *per capita* GDP of China. We considered the subgroups of patients of different age (<65 or ≥65), sex (male or female), ECOG performance status score (0 or 1), and disease status: Metastatic and Recurrent only (Supplementary Table S4).

## 3 Results

### 3.1 Base-case

In the overall population, the finotonlimab-chemo group achieved the highest life years (LYs: 1.84) and quality-adjusted life years (QALYs: 1.42) at a lower total cost ($30,588.90), making it the most cost-effective option. In contrast, the cetuximab-chemo group had the lowest clinical benefits (LYs: 1.32, QALYs: 1.00) despite a similar cost ($30,521.52). Compared to cetuximab-chemo, pembrolizumab monotherapy (ICER: 85,131.70/QALY) and pembrolizumab-chemo (ICER: 203,545.22/QALY) were less cost-effective, while finotonlimab-chemo (ICER: 161.13/QALY) was significantly more favorable. The NMB analysis supported this finding, with finotonlimab-chemo group having the highest INMB ($4,746.03) compared to cetuximab-chemo, followed by pembrolizumab ($-17,381.75) and pembrolizumab-chemo ($-32,841.18).

In the PD-L1 CPS ≥1 subgroup, cetuximab-chemo remained the least cost-effective, with the lowest QALYs (1.30) and a negative NMB ($-25,122.93). Pembrolizumab and pembrolizumab-chemo showed extremely high ICERs (2,537,841.42/QALY and 855,822.79/QALY, respectively), indicating poor cost-effectiveness. Similarly, in the PD-L1 CPS ≥20 subgroup, cetuximab-chemo again had the worst outcomes (QALYs: 1.06, NMB: $-21,776.00). Pembrolizumab monotherapy (QALY: 1.60) and pembrolizumab-chemo (QALY: 1.43) were more effective but at substantially higher costs, further reinforcing finotonlimab-chemo as the dominant strategy in the total population.

Overall, finotonlimab-chemo consistently demonstrated superior cost-effectiveness, particularly in the total population, whereas pembrolizumab-based regimens showed limited economic value in subgroups with higher PD-L1 expression (Table 3).

**TABLE 3:** Results of base-case.

Group	Total cost ($)	LYs	QALYs	ICER vs cetuximab-chemo ($/QALY)	NMB ($)	INMB vs cetuximab-chemo ($)
Overall population						
Cetuximab-chemo group	30,521.52	1.32	1.00		−18,488.13	
Pembrolizumab group	50,352.30	1.61	1.24	85,131.70	−35,869.89	−17,381.75
Pembrolizumab-chemo group	64,604.18	1.56	1.17	203,545.22	−51,329.31	−32,841.18
Finotonlimab-chemo group	30,588.90	1.84	1.42	161.13	−13,742.11	4,746.03
PD-L1 CPS ≥ 1						
Cetuximab-chemo group	40,389.46	1.73	1.30		−25,122.93	
Pembrolizumab group	57,392.11	1.70	1.31	2,537,841.42	−42,560.20	−17,437.27
Pembrolizumab-chemo group	71,558.43	1.79	1.34	855,822.79	−56,650.59	−31,527.66
PD-L1 CPS ≥ 20						
Cetuximab-chemo group	34,149.10	1.40	1.06		−21,776.00	
Pembrolizumab group	66,874.09	2.09	1.60	60,965.21	−48,352.29	−26,576.29
Pembrolizumab-chemo group	78,038.58	1.91	1.43	120,576.95	−61,742.70	−39,966.70

### 3.2 One-way sensitivity analysis

**FIGURE 2:**
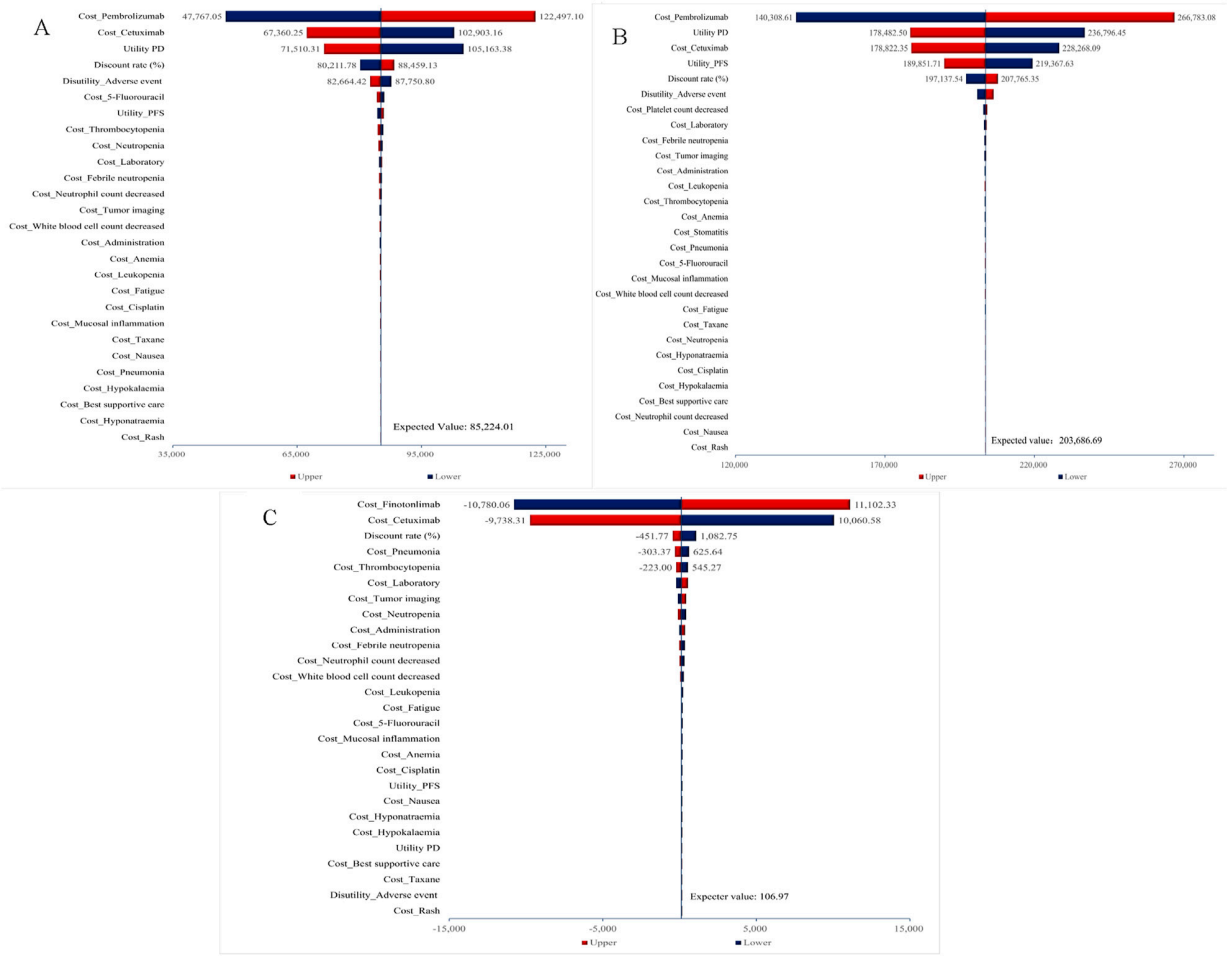
One-way sensitivity analysis for total population. **(A)** Pembrolizumab vs. Cetuximab-Chemo group. **(B)** Pembrolizumab-Chemo vs. Cetuximab-Chemo group. **(C)** Finotonlimab-Chemo vs. Cetuximab-Chemo group.

Figure 2 displayed tornado diagrams illustrating the results of the one-way sensitivity analysis from the overall population analysis. Drug costs were determined to be the most influential parameter affecting the ICER values, followed by the discount rate and utility values associated with PFS, PD, and adverse events. Under extreme conditions, the ICER values for pembrolizumab (monotherapy or combination) vs. cetuximab-chemo consistently surpass the WTP threshold of three times the *per capita* GDP, whereas the ICERs of finotonlimab-chemo vs. cetuximab-chemo remains below the WTP threshold, thereby demonstrating the robustness of the mode.

As shown in Supplementary Figure S7, for cases with PD L1 CPS≥1, utility PD remained the most critical parameter affecting the ICERs of pembrolizumab-chemo, followed by the cost of pembrolizumab and the health utility value of PFS state. The disutility of adverse events had the most significant impact on the ICERs for pembrolizumab, followed by the health utility value of PD state and PFS. In addition to the aforementioned three influencing factors for pembrolizumab group, the ICER values of pembrolizumab either as monotherapy or in combination all exceeded the WTP threshold under the influence of other parameters. The model demonstrated essential stability.

For cases with PD L1 CPS ≥20, the drug cost of pembrolizumab and cetuximab became the primary influencing factor in the monotherapy group. In the pembrolizumab combination therapy group, the impact of pembrolizumab cost, the health utility value of PD and the cost of cetuximab had notable effects on the outcome. Under extreme conditions, the ICER values for both pembrolizumab monotherapy and pembrolizumab-chemo groups consistently surpass the WTP threshold of three times the *per capita* GDP ($36,890.1) (Supplementary Figure S8).

### 3.3 Probabilistic sensitivity analysis

**FIGURE 3:**
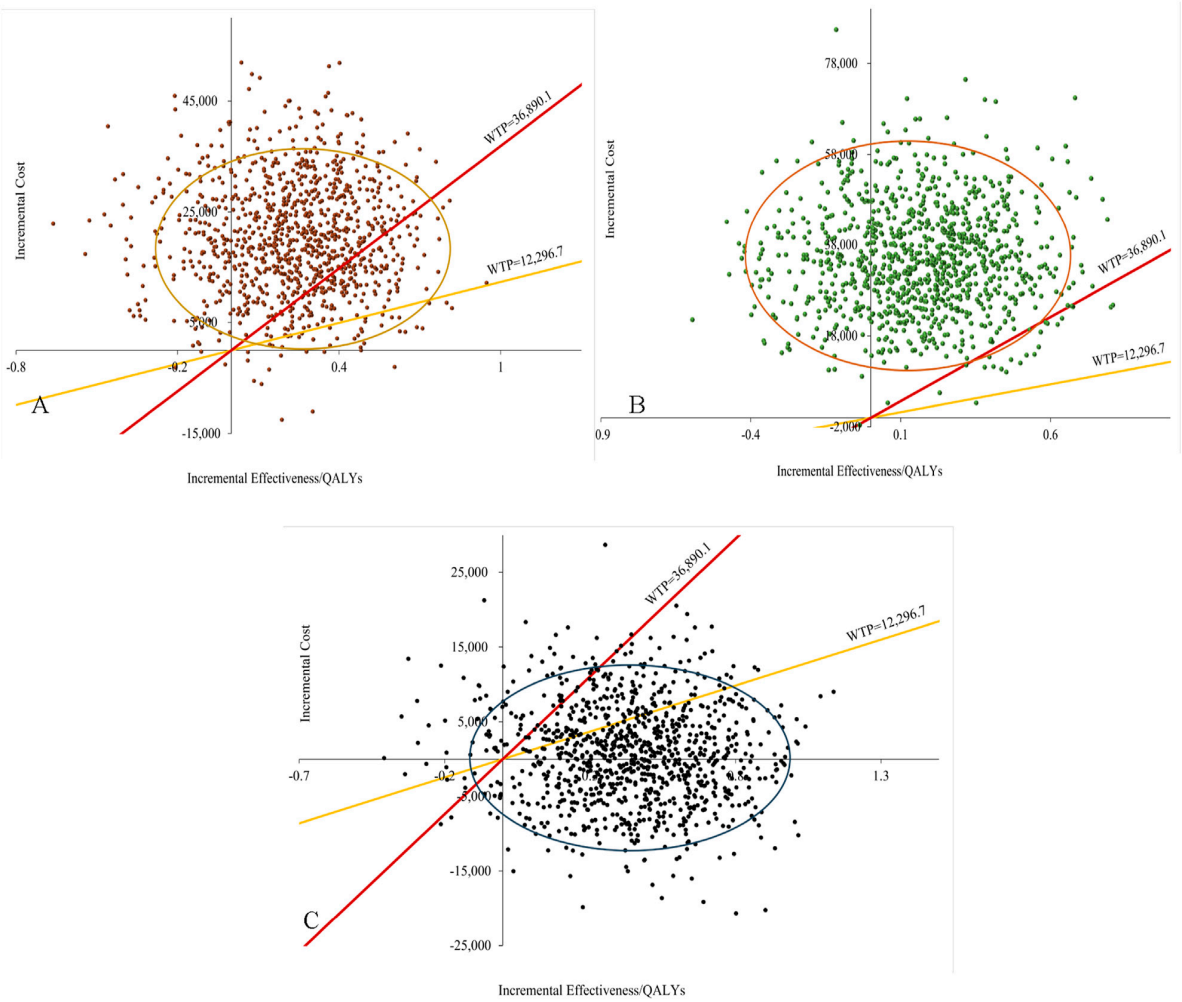
Probabilistic sensitivity analysis for total population. **(A)** Pembrolizumab vs. Cetuximab-Chemo group. **(B)** Pembrolizumab-Chemo vs. Cetuximab-Chemo group. **(C)** Finotonlimab-Chemo vs. Cetuximab-Chemo group.

**FIGURE 4:**
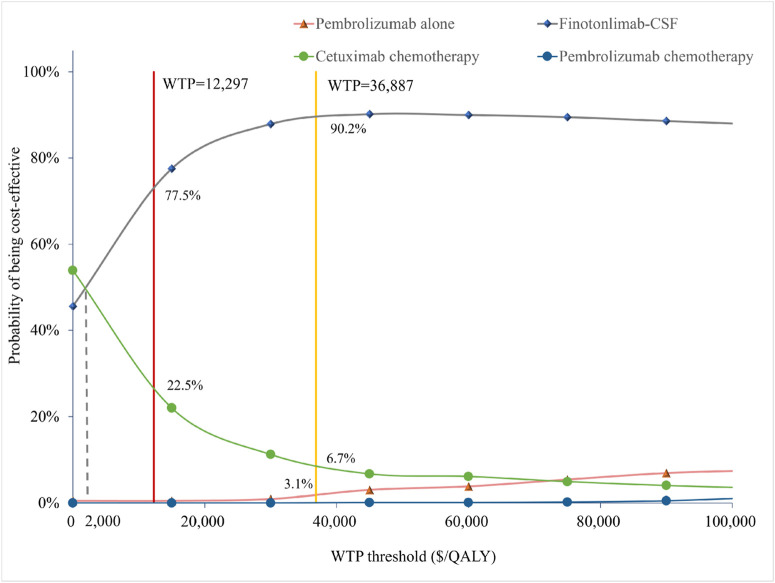
Cost-effectiveness acceptability curves of the different treatments.

The results of the probabilistic sensitivity analysis, including the cost-effectiveness scatter plot and the acceptability curve, are illustrated in Figures 3, 4, as well as in to Supplementary Figure S9-S11. Figures 3A,B demonstrated that, within the overall population, the majority of scatter points were positioned in the first quadrant and above the WTP threshold line. This suggested that, compared to the combination of cetuximab-chemo, pembrolizumab monotherapy or combination therapy exhibited superior efficacy but was also associated with higher costs, thereby failing to provide a cost-effectiveness advantage. The scatter plots depicting the populations with PD-L1 CPS ≥1 and PD-L1 CPS ≥20 exhibited notable similarities, as illustrated in Supplementary Figure S9-S10. Figure 3C demonstrated that, within the overall population, scatter points were uniformly distributed in the first and fourth quadrants. However, the majority of these points did not exceed the WTP threshold line, indicating that the finotonlimab-chemo exhibited a more pronounced cost-effectiveness superiority compared to the cetuximab-chemo.

Figure 4 illustrated that, within the total population, the economic probability of cetuximab-chemo decreased rapidly while that of finotonlimab-chemo increased rapidly when the WTP threshold exceeded $2,000 per QALY. The finotonlimab-chemo group demonstrated a 77.5% and 90.2% probability of being cost-effective at WTP thresholds of $12,297 and $36,887/QALY, respectively. Within the population with PD-L1 CPS ≥1 and CPS ≥20, as the WTP threshold increased, the probability of cetuximab being cost-effective rapidly decreased, while both pembrolizumab monotherapy and combination therapy demonstrated significant improvement in cost-effectiveness. When the WTP threshold was set at $36,887 per QALY, pembrolizumab monotherapy demonstrated a 10.8% and 21.9% probability of being cost-effective within the population with PD-L1 CPS ≥1 and CPS ≥20, respectively (Supplementary Figure S11).

### 3.4 Value of information analysis

**FIGURE 5:**
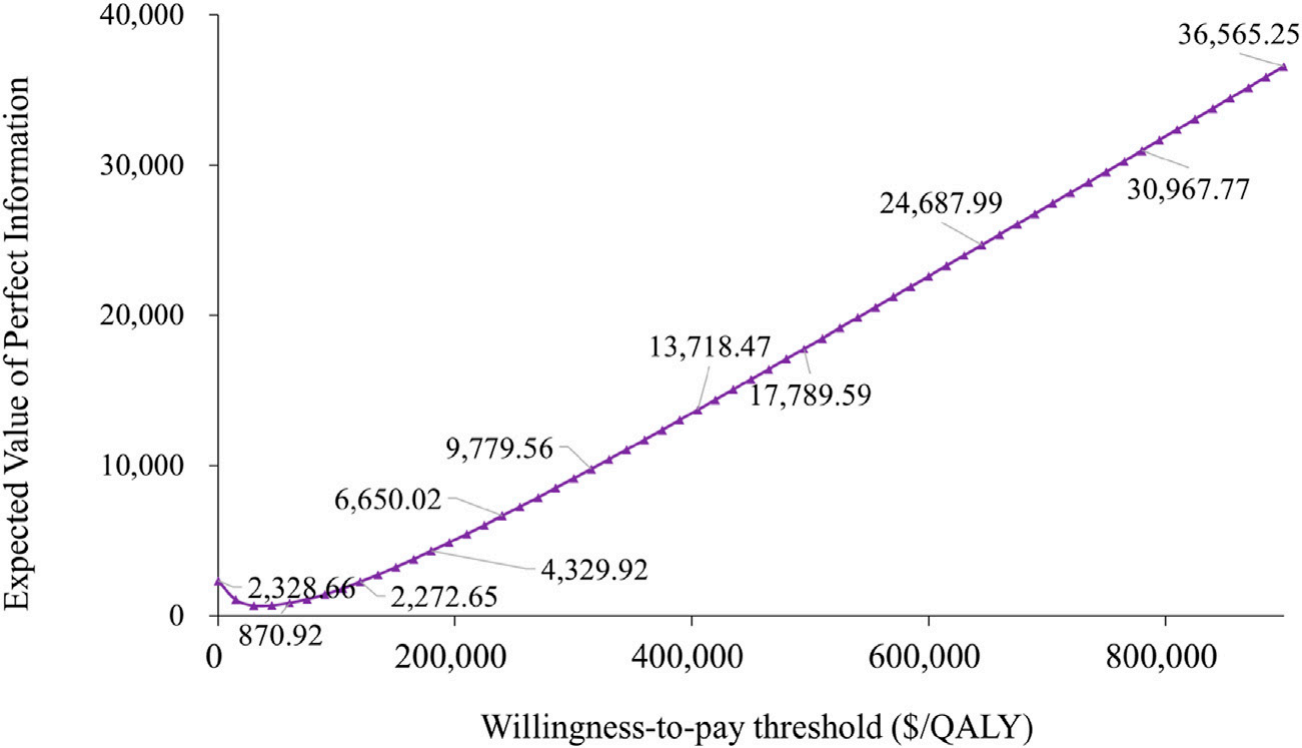
Expected value of perfect information curve.

Our analysis employing the Expected Value of Perfect Information (EVPI) approach demonstrated substantial variability in determining the optimal WTP threshold. At lower WTP thresholds, the EVPI started off low, with only $867.54 at a $20,000/QALY threshold. However, as the WTP threshold increased, the EVPI initially decreased, reaching a low point of $656.18, indicating that the value of obtaining more information diminished within certain threshold ranges. Subsequently, as the WTP threshold continued to rise, the EVPI increased significantly, showing a trend of falling and then rising. At a $200,000/QALY threshold, the EVPI was $5,076.34, and at a $400,000/QALY threshold, the EVPI reached $13,494.13. The findings demonstrate that variations in the cost-effectiveness threshold substantially influenced the economic evaluation outcomes, highlighting this parameter as a key sensitivity factor,and the value of obtaining more information also increased with the increase in the WTP threshold, demonstrating the dependency of the EVPI growth trend on the WTP threshold. This trend of falling and then rising further emphasized the dynamic changes in the value of information at different WTP thresholds (Figure 5).

### 3.5 Scenario analysis results

To address methodological and model uncertainties, we performed four scenario analyses. In the first scenario analysis, we evaluated the impact of cost variations by applying a ±20% adjustment to the estimated costs of finotonlimab, pembrolizumab, and cetuximab across health states. These adjusted cost parameters were then incorporated into the model to reassess the cost-effectiveness of each treatment regimen. Compared to cetuximab-chemotherapy, finotonlimab-chemotherapy remained more cost-effective than both pembrolizumab monotherapy and combined therapy, with an ICER of 1,202.89/QALY at +20% cost adjustment and −880.62/QALY at −20% cost adjustment, respectively. In scenario analysis 2, the treatment duration for pembrolizumab was extended by 3 months to align with real-world clinical scenarios. Similarly, finotonlimab retained its cost advantage, yielding the favorable ICER (161.13/QALY) and the highest INMB ($402,123.92). The results are presented in Table 4. In the third scenario analysis, we simulated a 60% price reduction for finotonlimab upon its anticipated NRDL inclusion in 2025 (year 2), and for both pembrolizumab and cetuximab starting in 2028 (year 4) due to patent expiration and intensified market competition, respectively. Since the treatment regimen for pembrolizumab is no longer administered in the fourth year, there was no impact compared to the base case analysis. The results still showed that finotonlimab maintained its cost advantage, yielding a favorable ICER (−8,769.61/QALY) and a better incremental net monetary benefit (INMB, $103.07). We also extended the time horizon to 30 years. The results remained consistent with the base-case analysis, with finotonlimab-chemotherapy demonstrating sustained cost-effectiveness (total cost: $34,752.22; QALYs: 1.42; ICER: $10,052.72 vs. cetuximab-chemo). The results are presented in Table 4.

**TABLE 4:** Results of the scenario analysis.

Group	Total cost ($)	LYs	QALYs	ICER vs cetuximab-chemo ($/QALY)	NMB ($)	INMB vs cetuximab-chemo ($)
Scenario 1 (+20%) - overall population						
Cetuximab-chemo group	34,661.24	1.32	1.00		−22,819.32	
Pembrolizumab group	59,056.18	1.61	1.24	104,725.19	−44,902.52	−22,083.20
Pembrolizumab-chemo group	75,192.93	1.56	1.17	242,059.46	−62,352.25	−39,532.93
Finotonlimab-chemo group	35,164.26	1.84	1.42	1,202.89	−18,566.70	4,252.61
Scenario 1 (−20%) - overall population						
Cetuximab-chemo group	26,381.79	1.32	1.00		−14,367.68	
Pembrolizumab group	41,648.42	1.61	1.24	65,538.22	−26,646.39	−12,278.71
Pembrolizumab-chemo group	54,015.43	1.56	1.17	165,030.98	−40,194.05	−25,826.37
Finotonlimab-chemo group	26,013.54	1.84	1.42	−880.62	−8,792.83	5,574.85
Scenario 2 - overall population						
Cetuximab-chemo group	30,521.52	1.32	1.00		895,558.02	
Pembrolizumab group	52,353.40	1.61	1.24	93,722.25	1,094,876.98	199,318.96
Pembrolizumab-chemo group	66,545.48	1.56	1.17	215,138.84	1,011,326.20	115,768.18
Finotonlimab-chemo group	30,588.90	1.84	1.42	161.13	1,297,681.94	402,123.92
Scenario 3 - dynamic price adjustment						
Cetuximab-chemo group	30,284.83	1.32	1.00		−9,791.60	8,696.53
Finotonlimab-chemo group	26,851.86	1.84	1.42	−8,769.61	−18,385.06	103.07
Scenario 4–30 years adjustment						
Cetuximab-chemo group	32,152.93	1.32	1.00	610,280.57	−20,746.20	−2,258.07
Pembrolizumab group	52,282.67	1.61	1.24	92,358.70	−38,120.32	−19,632.19
Pembrolizumab-chemo group	65,043.08	1.56	1.17	202,926.65	−51,083.99	−32,595.86
Finotonlimab-chemo group	34,752.22	1.84	1.42	10,052.72	−19,361.82	−873.69

### 3.6 Subgroup analysis

Results of the subgroup analysis are presented in Supplementary Table S5. At a WTP threshold of $12,296.7/QALY, the subgroup with the highest probability of being cost-effective was the Age category ≥65 years finotonlimab-chemo subgroup with a cost of 31,613.62, an ICER of 1,740.88 (compared to cetuximab-chemotherapy group), followed by the subgroup with a baseline ECOG performance status of 0 finotonlimab-chemo subgroup with a cost of 31,818.07, an ICER of 1,942.91 (compared to cetuximab-chemotherapy group). A similar trend was observed at a WTP threshold of 3 times the *per capita* GDP of China.

## 4 Discussion

Significant advancements have been achieved in the first-line treatment regimens for R/M HNSCC in recent years (Bhatia and Burtness, 2023; Ghosh et al., 2022), particularly with the introduction of ICIs, which have substantially improved patient prognoses. Nevertheless, the prohibitively high cost of these innovative therapies imposes a considerable financial burden on both patients and the healthcare system. Previous studies have investigated the cost-effectiveness of cetuximab (Lang and Dong, 2020) and pembrolizumab (Lang et al., 2020) for the first-line treatment of R/M HNSCC. Considering the substantial expense of these drugs and the relatively low *per capita* income in China, none of the aforementioned treatment regimens appear to be cost-effective from a Chinese perspective. The remarkable efficacy and safety profile of the domestically developed novel drug, finotonlimab (Shi et al., 2024), have disrupted the monopoly of imported drugs in the first-line treatment of R/M HNSCC, thereby exerting a significant and lasting impact on the industry. This study revealed that finotonlimab demonstrated a 90.2% probability of being cost-effective at a WTP threshold of $36,887 per QALY. As market competition intensifies or medical insurance negotiations progress, the price of finotonlimab may decrease further, and the economic advantage of finotonlimab will be further enhanced. Furthermore, our research indicated that, when compared with other commonly utilized first-line treatment regimens in China, the combination of finotonlimab and chemotherapy exhibited the highest INMB value and the greatest cost-effectiveness superiority. The introduction of finotonlimab not only offers novel therapeutic alternatives for patients with R/M HNSCC but also, given its remarkable cost-effectiveness, is expected to reshape the current treatment landscape.

The KEYNOTE-048 study (Burtness et al., 2019) indicated that patients with PD-L1 CPS ≥20 and CPS ≥1 exhibited significantly greater OS benefits compared to the overall population. Similarly, finotonlimab research (Shi et al., 2024) indicated that, when compared to chemotherapy alone, the finotonlimab-chemotherapy decreased the risk of mortality by 50% in patients with PD-L1 CPS ≥20 and by 27% in patients with PD-L1 CPS ≥1. Unfortunately, the finotonlimab treatment could not be included in the cost-effectiveness subgroup analysis, as data regarding the impact of PD-L1 CPS expression on PFS were not available for finotonlimab. Our research indicated that, in both the total population and the subgroups with PD-L1 CPS ≥20 or CPS ≥1, neither pembrolizumab monotherapy nor pembrolizumab combination therapy demonstrated economic superiority over cetuximab-chemotherapy. The ICER values consistently exceeded the WTP threshold, as confirmed by both univariate sensitivity analysis and probabilistic sensitivity analysis. These results are consistent with those of Lang’s research (Lang et al., 2020), but inconsistent with the findings of Zhou et al. (2020). This might be due to the fact that the data of Zhou et al. (2020) were derived from the real world. Furthermore, our research indicated that in populations with CPS ≥20 and all population, pembrolizumab monotherapy demonstrated greater cost-effectiveness compared to pembrolizumab combination therapy. This may be attributed to the price competitiveness and superior safety profile of the pembrolizumab monotherapy group. Our findings on the cost-effectiveness of finotonlimab-chemotherapy were further reinforced under a scenario of dynamic price adjustment. Assuming finotonlimab enters the NRDL in 2025, its earlier price reduction would solidify its position as the most cost-effective strategy, even in the face of expected future price competition for pembrolizumab and cetuximab.

The WTP threshold employed in this analysis (1–3 times China’s *per capita* GDP) follows a conventional framework often applied in health economic evaluations, particularly in China (Zhang et al., 2025; Wang et al., 2025). This range, while criticized for its simplicity and lack of direct empirical foundation in health opportunity costs, remains widely used in global health policy assessments, including those by the WHO, as it provides a pragmatic benchmark that is tethered to a country’s economic capacity (Kazibwe et al., 2022). Furthermore, emerging research by Xu et al. suggests a more precise empirical WTP threshold of 1.94 times *per capita* GDP for end-stage diseases in China (Xu L. et al., 2024). At this specific threshold, our probabilistic sensitivity analysis indicates that finotonlimab-chemotherapy demonstrates an 83% probability of being cost-effective, compared to 17% for cetuximab-chemotherapy. This finding further reinforces the robustness of our conclusion regarding the economic value of finotonlimab-based therapy, even under a more stringent, empirically-derived WTP benchmark.

There are some limitations of this study: 1) The clinical data were derived from a pooled analysis of three Phase III trials rather than a single clinical trial. While this data integration approach allowed for the consolidation of information from multiple studies, potential differences among the trials in terms of design, inclusion criteria, and implementation environment may introduce heterogeneity into the patient-level data. 2) These data were not collected directly at the individual patient level but were based on aggregated and analyzed overall data, which may not fully capture the detailed characteristics of individual patients. In real-world clinical practice, eligibility for subsequent treatment is influenced by multiple factors. Particularly for patients with poor performance status, they are more likely to opt for best supportive care rather than active anti-cancer therapy. 3) In the real world, factors related to payments (such as out-of-pocket expenses for patients) are not fully reflected in the model. At the same time, there may be uncertainties in the model structure and the sources of parameters. These limitations may have some impact on the accuracy of the economic evaluation results of the treatment options. 4) The clinical and economic inputs for our model were primarily derived from RCTs, which, while methodologically rigorous, may not fully capture the heterogeneity, compliance patterns, and long-term outcomes of a real-world patient population. 5) Future research would greatly benefit from incorporating real-world evidence from Chinese oncology databases to validate our model’s predictions, particularly regarding treatment patterns after progression, the management of adverse events in routine care, and overall survival in unselected patients. 6) Our model did not account for the potential impact of common patient comorbidities (e.g., cardiovascular disease, diabetes) on utility values, treatment adherence, or costs. The absence of stratified data from the source trials precluded such analysis. Finally, our analysis adopted a national payer perspective and did not evaluate the potential variation in cost-effectiveness across different healthcare settings in China. Differences in unit costs, local treatment patterns, and real-world outcomes could influence the economic evaluation results. Generating such setting-specific evidence requires detailed local cost and outcome data, which represents an important direction for future research. Future studies incorporating real-world data could provide further insights into how comorbid conditions influence the cost-effectiveness of treatment strategies for R/M HNSCC.

## 5 Conclusion

This study conducted a cost-effectiveness analysis of finotonlimab, pembrolizumab either monotherapy or combination therapy and cetuximab-chemotherapy from the perspective of Chinese payers. It addresses the gap in economic evidence for finotonlimab in the first-line treatment of R/M HNSCC. Furthermore, the indirect comparison between finotonlimab and currently prevalent first-line treatment regimens provides comprehensive economic evidence to support clinical decision-making, offering an essential reference for the dynamic adjustment of the medical insurance catalog and the development of rational drug use policies.

## Data Availability

The original contributions presented in the study are included in the article/Supplementary Material, further inquiries can be directed to the corresponding authors.
