# Supplementary material for: Cost-effectiveness analysis of first-line treatments for recurrent or metastatic head and neck cancer in China: an economic evaluation based on network meta-analysis
Source: Front Pharmacol. 2025 Sep 30;16:1644426. doi: 10.3389/fphar.2025.1644426 (PMC12517582; doi:10.3389/fphar.2025.1644426)
Supplement: Supplementary file 1 [file Supplementaryfile1.docx]

Supplementary


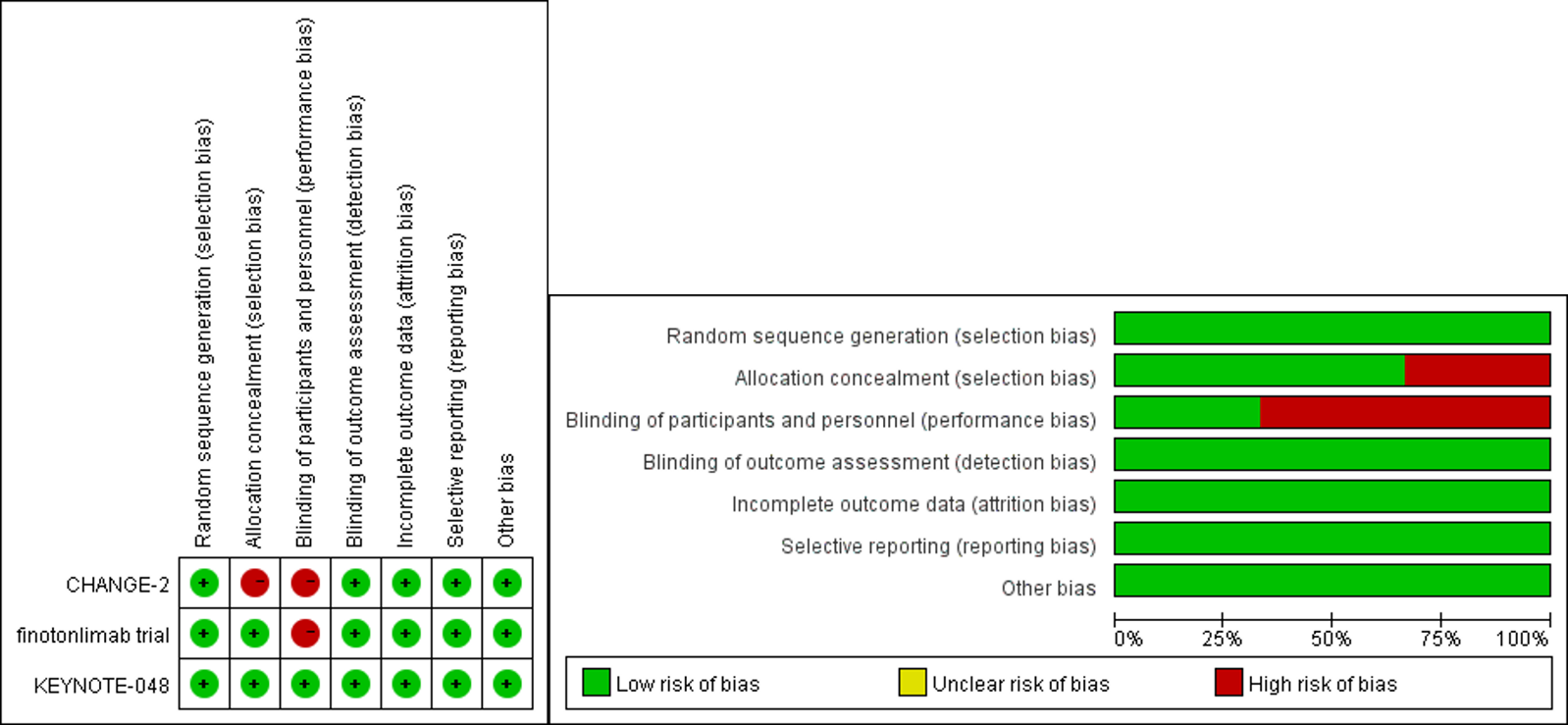


Figure S1 Summary of results from assessment of studies using the cochrane risk of bias tool.


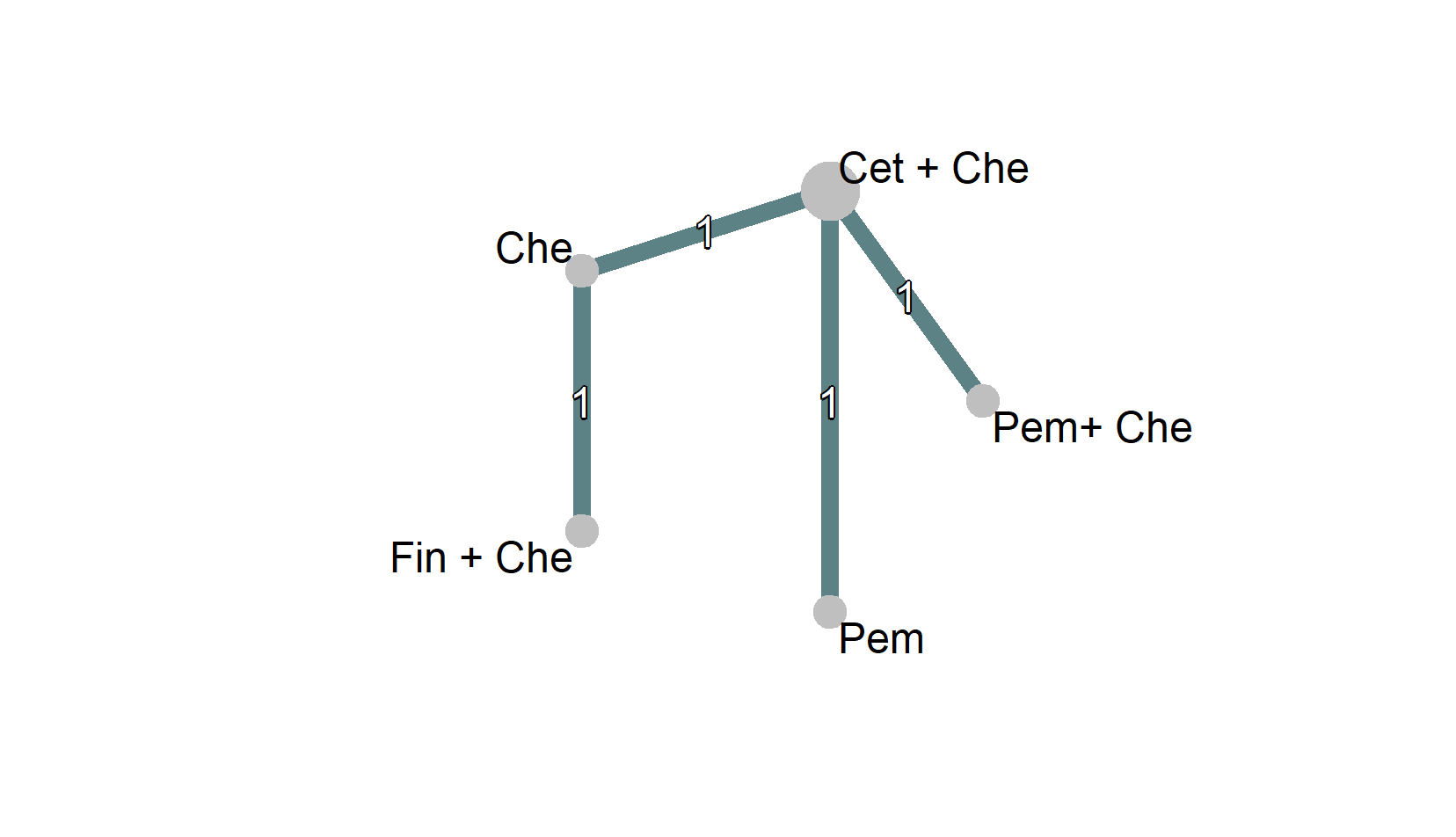


Figure S2 Model schematic for network meta-analysis. Che, chemotherapy; Cet, cetuximab; Pem, pembrolizumab; Fin, finotonlimab.


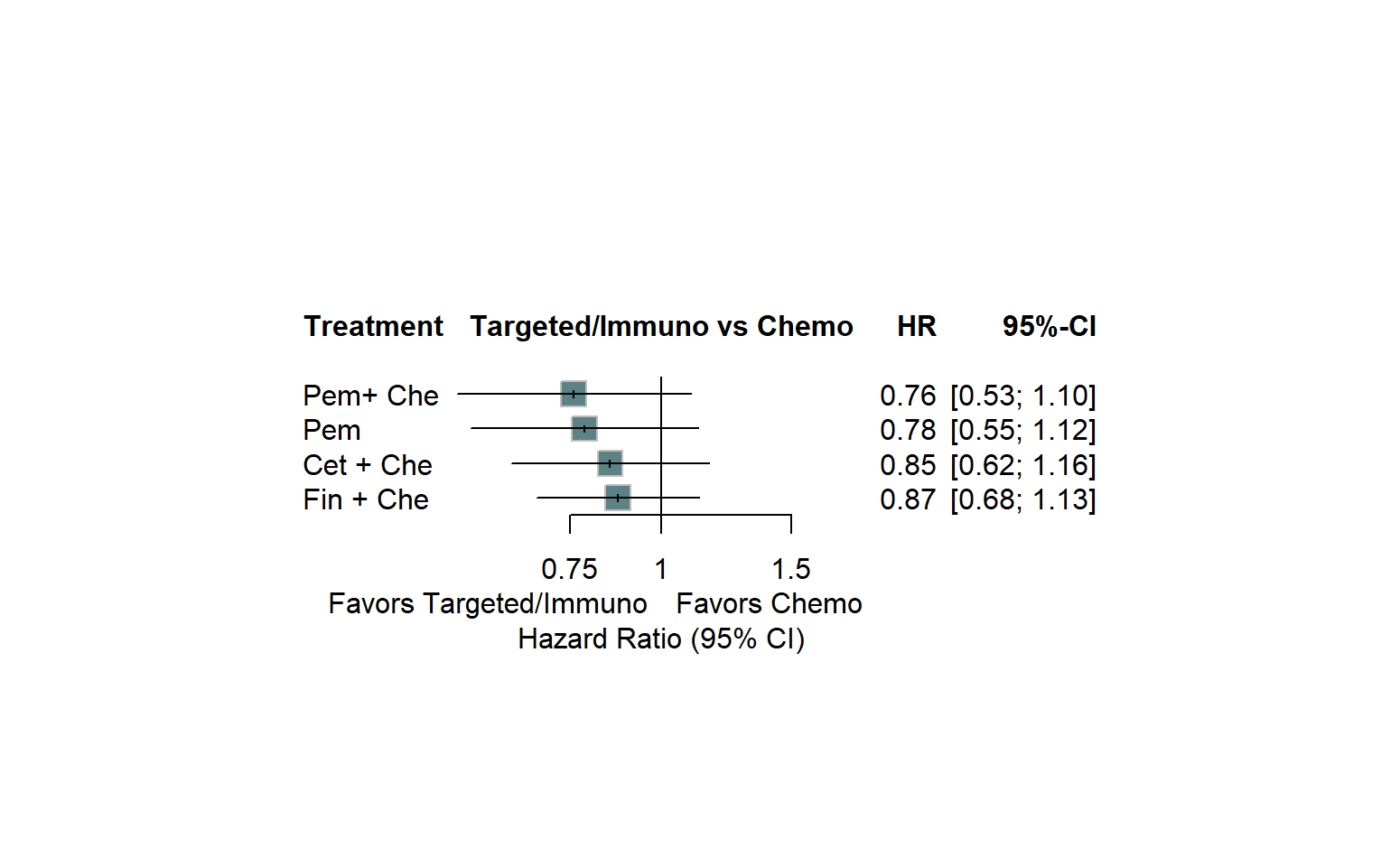


Figure S3 Summary of OS results from assessment of studies using the Cochrane Risk of bias tool. Che, chemotherapy; Cet, cetuximab; Pem, pembrolizumab; Fin, finotonlimab.


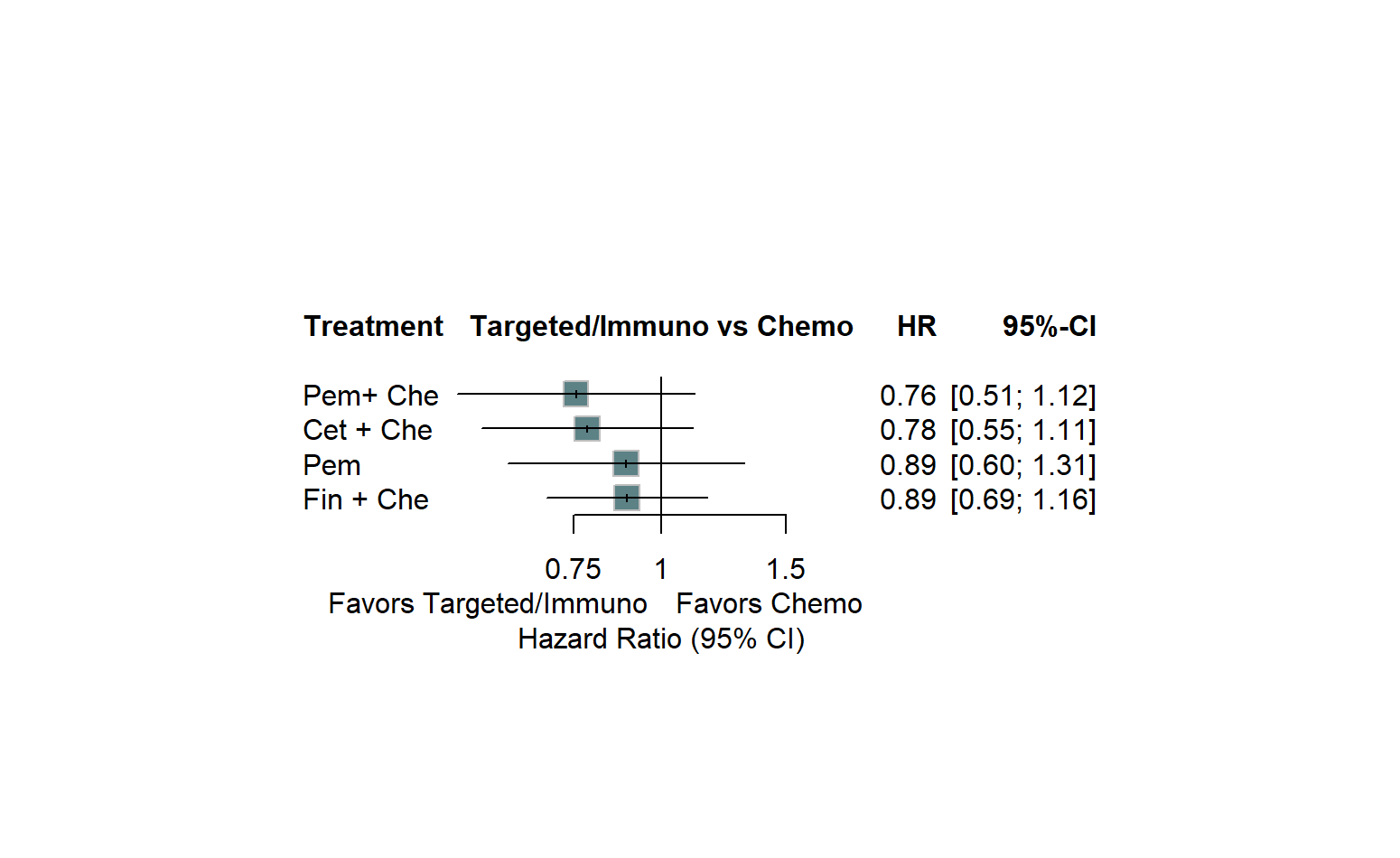


Figure S4 Summary of PFS results from assessment of studies using the Cochrane Risk of bias tool. Che, chemotherapy; Cet, cetuximab; Pem, pembrolizumab; Fin, finotonlimab.


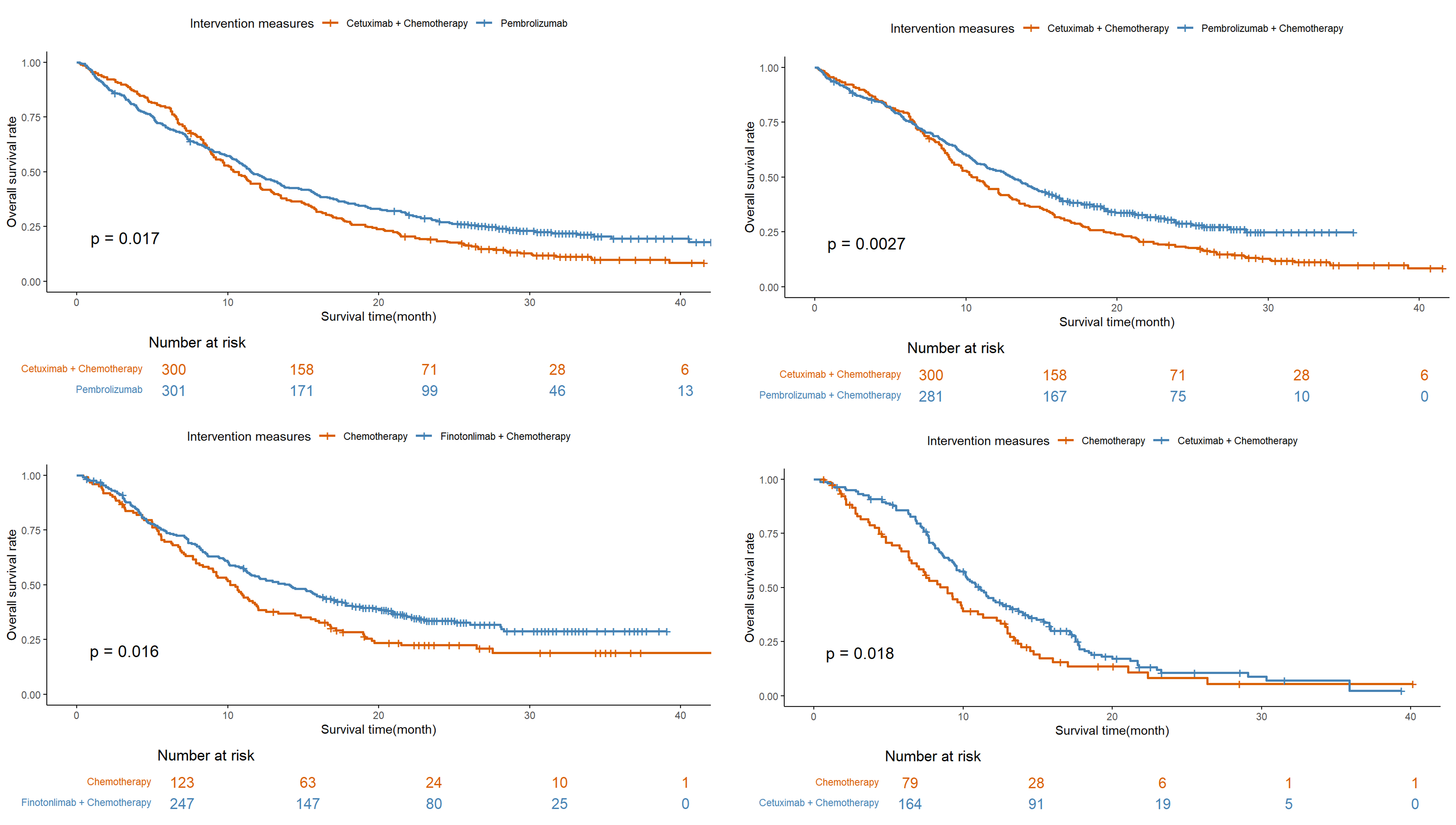


Figure S5 Original OS Curves of KEYNOTE-048, Finotonlimab trial, and CHANGE-2


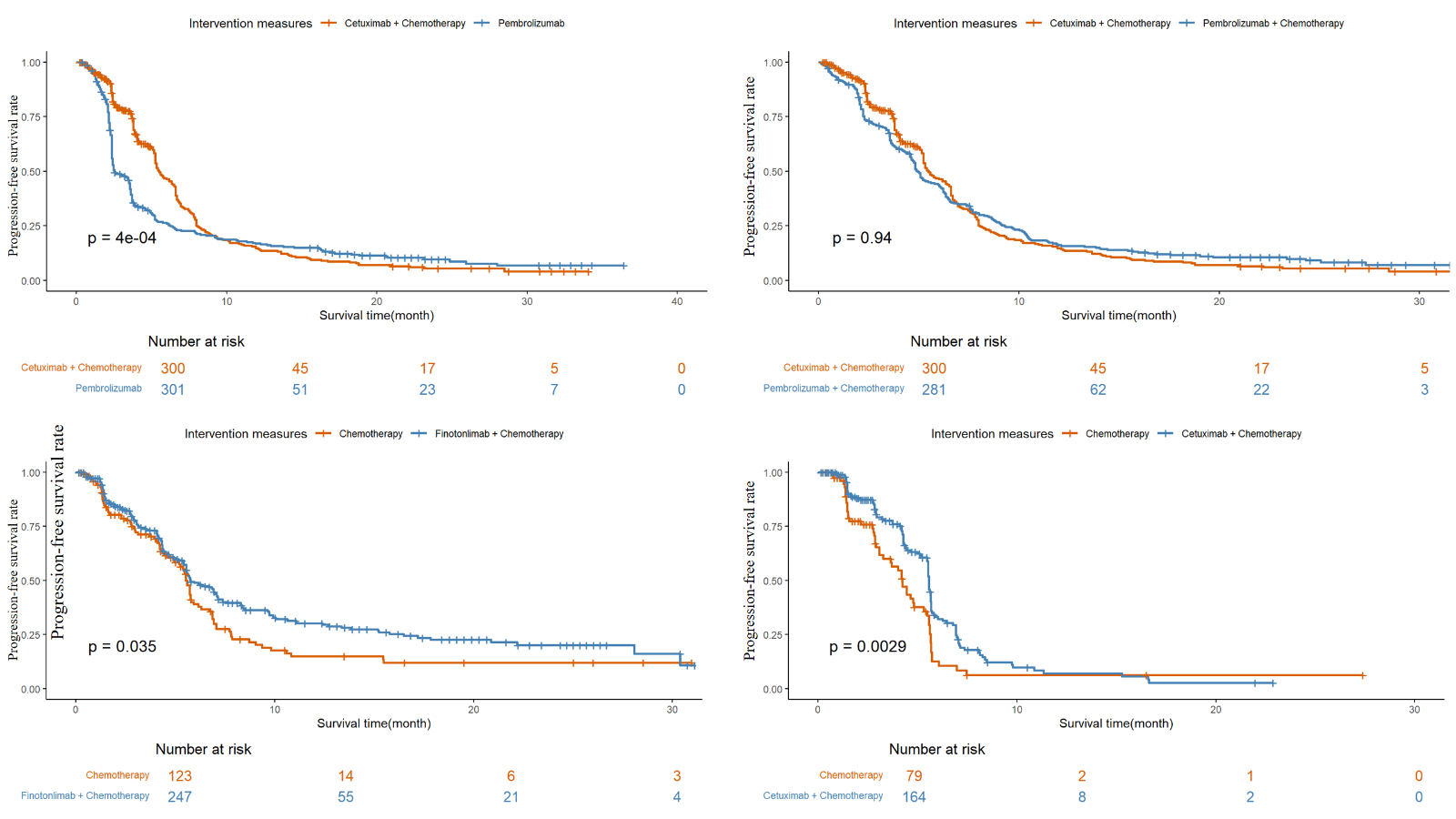


Figure S6 Original PFS Curves of KEYNOTE-048, Finotonlimab trial, and CHANGE-2


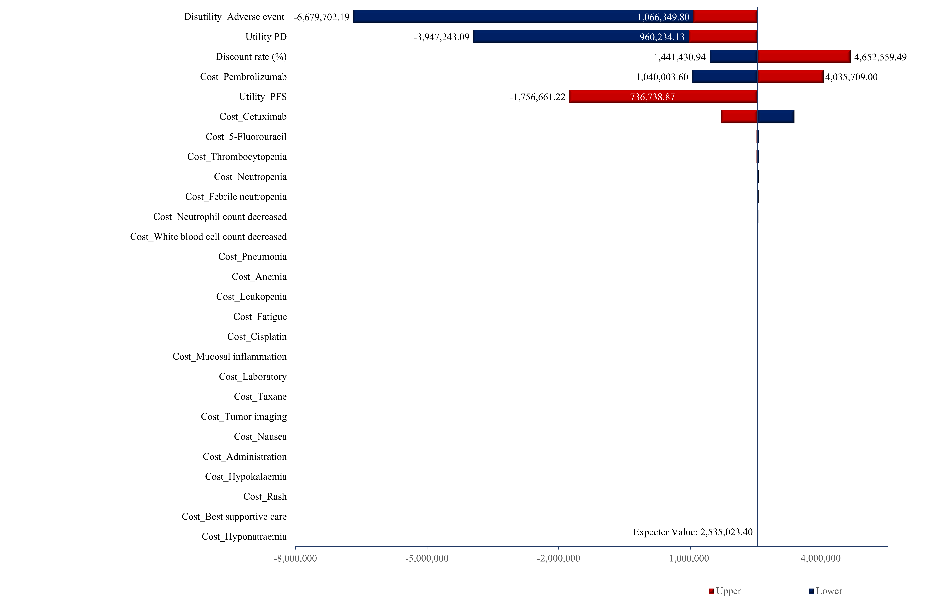


A


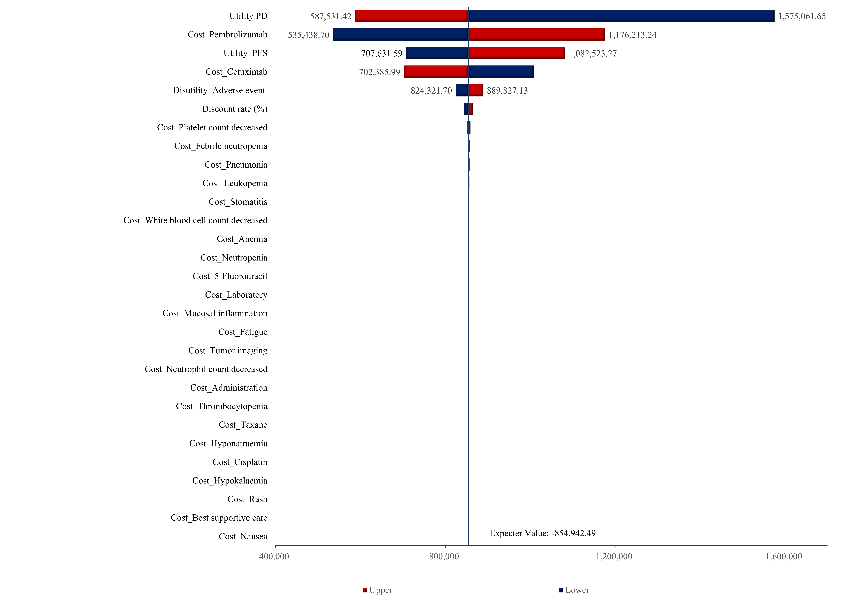


B

Figure S7 One-way sensitivity analysis for PD-L1 CPS≥1. (A) Pembrolizumab *vs.* Cetuximab-Chemo group. (B) Pembrolizumab-Chemo *vs.* Cetuximab-Chemo group.


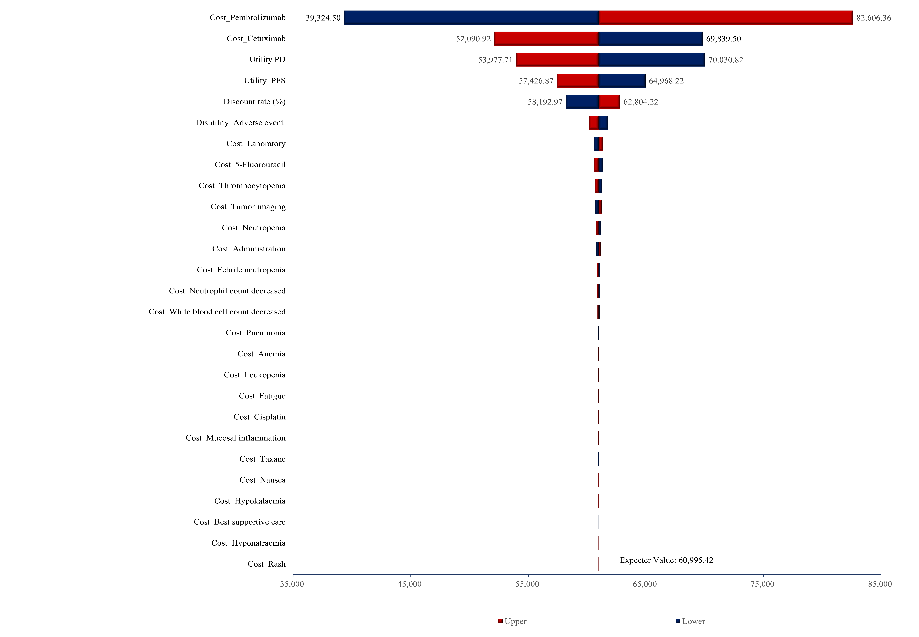


A


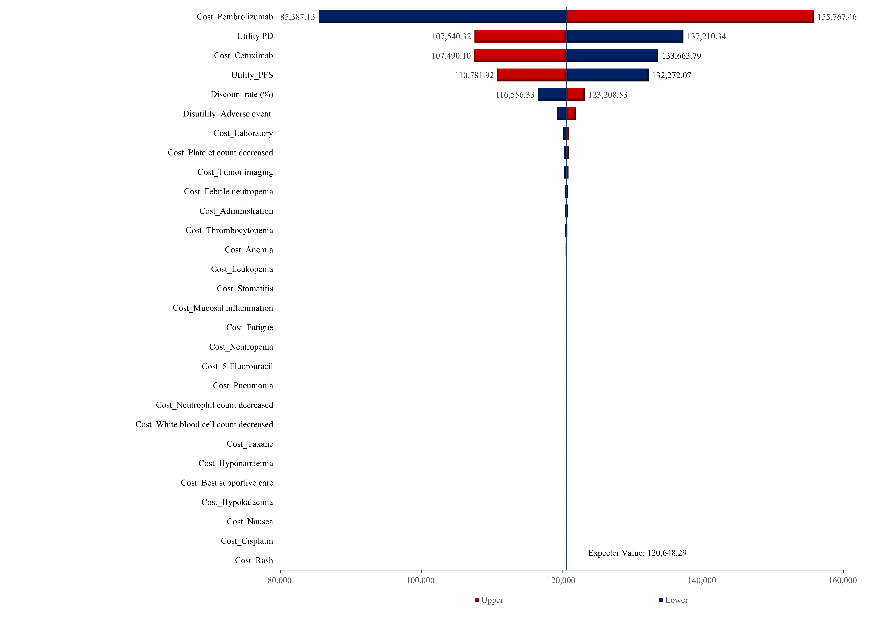


B

Figure S8 One-way sensitivity analysis for PD-L1 CPS≥20. (A) Pembrolizumab *vs.* Cetuximab-Chemo group. (B) Pembrolizumab-Chemo *vs.* Cetuximab-Chemo group.


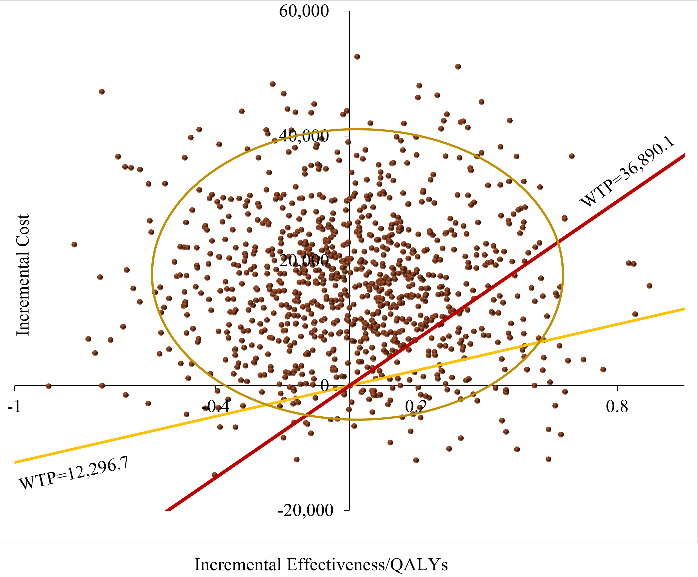


A


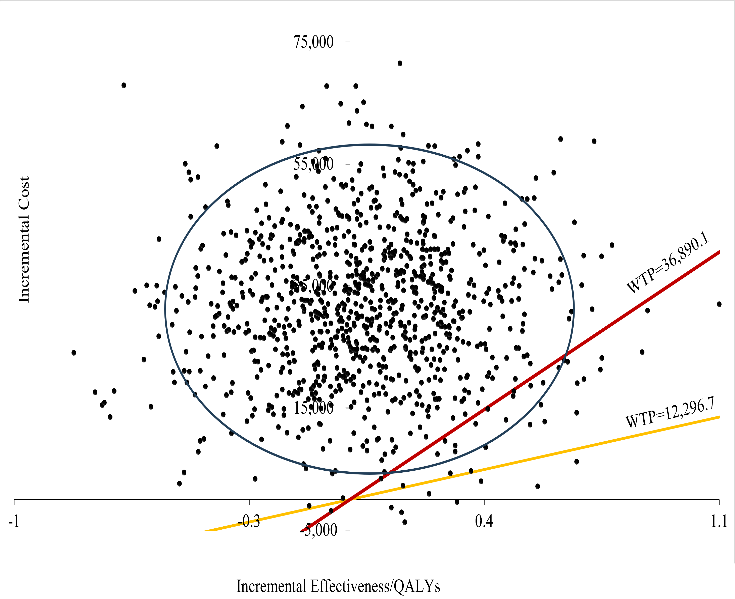


B

Figure S9 Probabilistic sensitivity analysis for PD-L1 CPS≥1. (A) Pembrolizumab vs. Cetuximab-Chemo group. (B) Pembrolizumab-Chemo vs. Cetuximab-Chemo group.


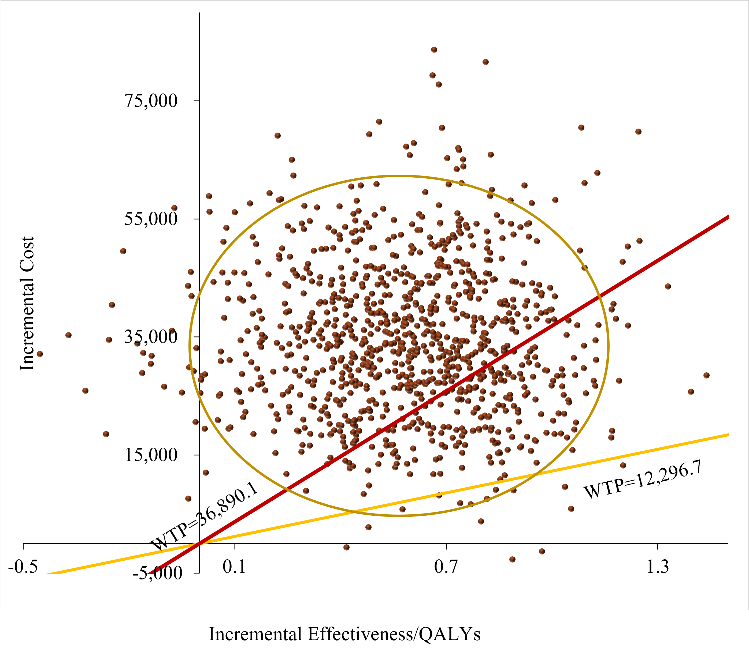


A


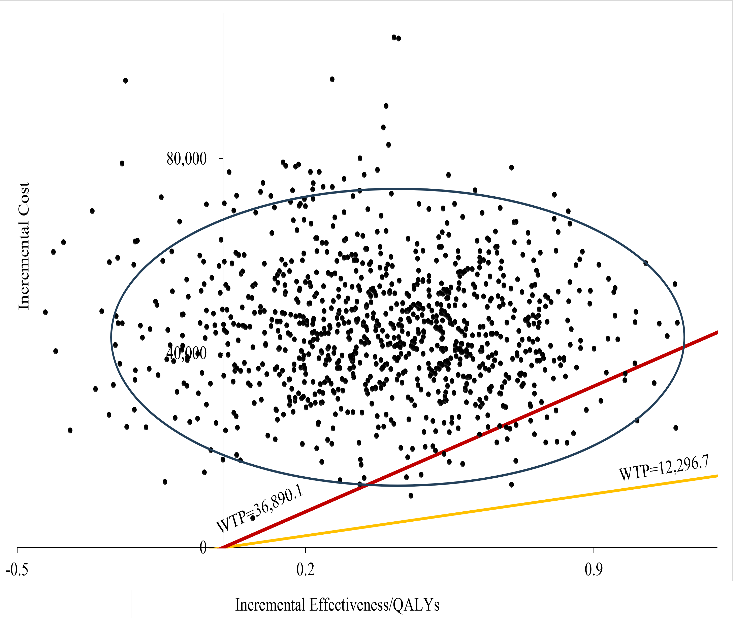


B

A

Figure S10 Probabilistic sensitivity analysis for PD-L1 CPS≥20. (A) Pembrolizumab vs. Cetuximab-Chemo group. (B) Pembrolizumab-Chemo vs. Cetuximab-Chemo group.


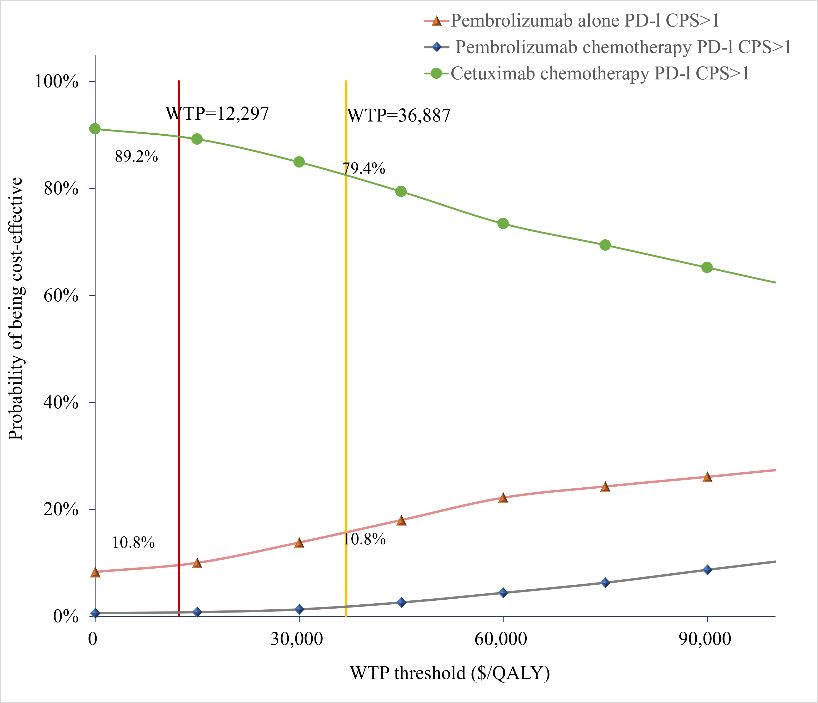


A


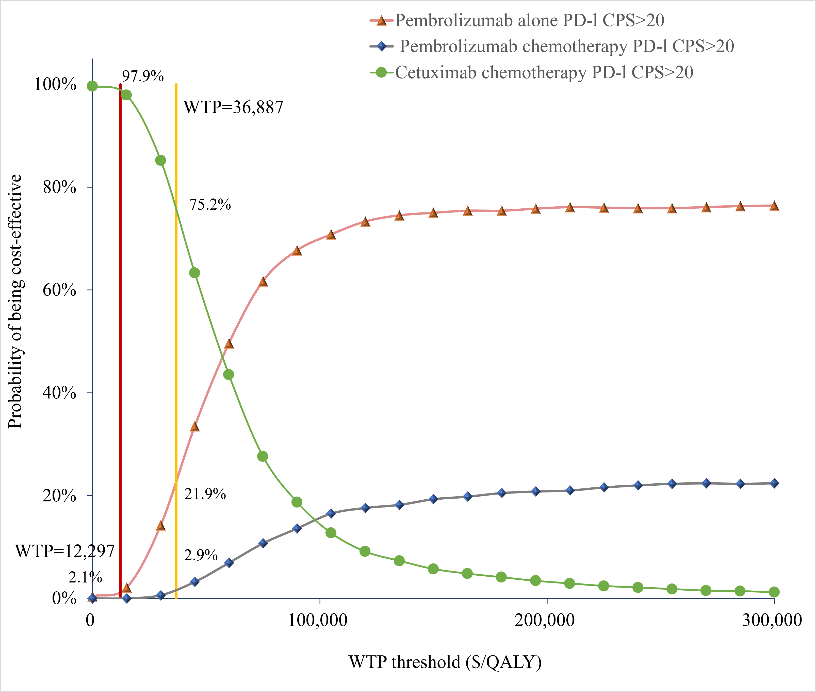


B

Figure S11 Cost-effectiveness acceptability curves of the different treatments for PD-L1 CPS≥1（A）and PD-L1 CPS≥20（B）.

**Table S1** Goodness-of-fit under different distributions of survival curves between the three patient groups.

| Distribution function | Fit metrics | Expotional | Gamma | Gengamma | Gompertz | Weibull | WeibullPH | Log-logistic | Log-normal |
| --- | --- | --- | --- | --- | --- | --- | --- | --- | --- |
| OS curve of the pembrolizumab | AIC | 1873.437 | 1875.054 | 1863.672 | 1867.788 | 1874.01 | 1874.01 | 1863.136 | 1862.472 |
|  | BIC | 1877.144 | 1882.468 | 1874.793 | 1875.202 | 1881.424 | 1881.424 | 1870.551 | 1869.886 |
| PFS curve of the pembrolizumab | AIC | 1522.368 | 1524.248 | 1347.63 | 1470.914 | 1520.394 | 1520.394 | 1404.828 | 1414.239 |
|  | BIC | 1526.075 | 1531.662 | 1358.751 | 1478.328 | 1527.809 | 1527.809 | 1412.242 | 1421.653 |
| OS curve of the pembrolizumab-chemotherapy | AIC | 1557.93 | 1559.541 | 1560.405 | 1559.593 | 1559.743 | 1559.743 | 1558.54 | 1567.934 |
|  | BIC | 1561.568 | 1566.818 | 1571.32 | 1566.87 | 1567.02 | 1567.02 | 1565.817 | 1575.211 |
| PFS curve of the pembrolizumab-chemotherapy | AIC | 1525.256 | 1525.305 | 1504.355 | 1517.674 | 1527.184 | 1527.184 | 1492.885 | 1503.819 |
|  | BIC | 1528.894 | 1532.582 | 1515.27 | 1524.951 | 1534.461 | 1534.461 | 1500.162 | 1511.095 |
| OS curve of the cetuximab- chemotherapy | AIC | 1983.408 | 1970.91 | 1971.558 | 1983.548 | 1973.695 | 1973.695 | 1967.571 | 1991.885 |
|  | BIC | 1987.111 | 1978.318 | 1982.669 | 1990.955 | 1981.103 | 1981.103 | 1974.979 | 1999.293 |
| PFS curve of the cetuximab- chemotherapy | AIC | 1501.707 | 1468.292 | 1438.649 | 1503.688 | 1484.456 | 1484.456 | 1424.945 | 1436.689 |
|  | BIC | 1505.411 | 1475.7 | 1449.76 | 1511.096 | 1491.864 | 1491.864 | 1432.353 | 1444.096 |
| OS curve of the finotonlimab- chemotherapy | AIC | 1333.823 | 1335.411 | 1323.084 | 1331.278 | 1335.82 | 1335.82 | 1322.472 | 1321.141 |
|  | BIC | 1337.332 | 1342.43 | 1333.612 | 1338.297 | 1342.839 | 1342.839 | 1329.491 | 1328.16 |
| PFS curve of the finotonlimab- chemotherapy | AIC | 1098.374 | 1099.286 | 1069.011 | 1091.572 | 1100.371 | 1100.371 | 1073.025 | 1070.469 |
|  | BIC | 1101.883 | 1106.305 | 1079.539 | 1098.591 | 1107.39 | 1107.39 | 1080.044 | 1077.488 |
| OS curve of the chemotherapy | AIC | 723.2055 | 723.8433 | 717.1502 | 723.1082 | 724.8576 | 724.8576 | 713.8117 | 715.1507 |
|  | BIC | 726.0176 | 729.4676 | 725.5867 | 728.7326 | 730.482 | 730.482 | 719.4361 | 720.7751 |
| PFS curve of the chemotherapy | AIC | 540.4595 | 539.8205 | 523.9936 | 536.9483 | 542.0244 | 542.0244 | 520.9185 | 523.0272 |
|  | BIC | 543.2716 | 545.4448 | 532.4301 | 542.5727 | 547.6488 | 547.6488 | 526.5429 | 528.6516 |
| OS curve of the cetuximab- chemotherapy | AIC | 990.4981 | 953.5008 | 955.0985 | 970.4365 | 954.1928 | 954.1928 | 954.6812 | 972.2771 |
|  | BIC | 993.598 | 959.7005 | 964.3981 | 976.6362 | 960.3925 | 960.3925 | 960.8809 | 978.4768 |
| PFS curve of the cetuximab- chemotherapy | AIC | 660.0145 | 604.2961 | 603.2487 | 647.142 | 615.1073 | 615.1073 | 597.3781 | 602.5625 |
|  | BIC | 663.1143 | 610.4959 | 612.5483 | 653.3417 | 621.307 | 621.307 | 603.5778 | 608.7623 |
| OS curve of the chemotherapy | AIC | 440.2644 | 433.1148 | 433.1992 | 440.9031 | 435.1601 | 435.1601 | 431.9339 | 431.8292 |
|  |  | 442.6339 | 437.8537 | 440.3075 | 445.642 | 439.899 | 439.899 | 436.6728 | 436.5681 |
| PFS curve of the chemotherapy | BIC | 287.2706 | 274.0339 | 265.4549 | 289.2666 | 280.8823 | 280.8823 | 262.8877 | 287.2706 |
|  |  | 289.64 | 278.7728 | 272.5633 | 294.0055 | 285.6212 | 285.6212 | 267.6266 | 289.64 |

**Table S2** Parameters of parametric models for virtual time-to-event data.

| Trial names | Treatment regimens | Endpoint | Distribution | Distribution information |
| --- | --- | --- | --- | --- |
| KEYNOTE-048 | Pembrolizumab | OS | Log-normal | meanlog: 2.43742  sdlog: 1.35058 |
|  |  | PFS | Generalised gamma | mu: 0.842358  sigma: 0.779814  Q: -1.224143 |
|  | Pembrolizumab-  chemotherapy | OS | Expotional | rate: 0.0503094 |
|  |  | PFS | Log-logistic | shape: 1.60275  scale: 5.01037 |
|  | Cetuximab-chemotherapy | OS | Log-logistic | shape: 1.73959  scale: 10.81143 |
|  |  | PFS | Log-logistic | shape: 2.15897  scale: 5.51996 |
| Finotonlimab trial | Finotonlimab-  chemotherapy | OS | Log-normal | meanlog: 2.66132  sdlog: 1.30498 |
|  |  | PFS | Log-normal | meanlog: 1.93456  sdlog: 1.17186 |
|  | Chemotherapy | OS | Log-logistic | shape: 1.51113  scale: 10.34558 |
|  |  | PFS | Log-logistic | shape: 1.70904  scale: 5.18547 |
| CHANGE-2 | Cetuximab- chemotherapy | OS | Gamma | shape: 2.1656  rate: 0.1591 |
|  |  | PFS | Log-logistic | shape: 2.76852  scale: 5.19973 |
|  | Chemotherapy | OS | Log-normal | meanlog: 2.062430  sdlog: 0.903392 |
|  |  | PFS | Log-normal | meanlog: 1.316805  sdlog: 0.745903 |

**Table S3** The treatment regimen and dosing schedule.

| First-line chemotherapy regimen | Dosing schemes |
| --- | --- |
| Cetuximab-Chemo group | cetuximab (400 mg/m² loading dose, then 250 mg/m² per week) until disease progression, intolerable toxicity, or  physician or participant decision +chemo^a^ |
| Pembrolizumab group | pembrolizumab 200 mg every 3 weeks for up to 35 cycles |
| Pembrolizumab-Chemo group | pembrolizumab 200 mg every 3 weeks for up to 35 cycles+chemo^a^ |
| Finotonlimab-Chemo group | Finotonlimab 200 mg every 3 weeks for up to 35 cycles+chemo^b^ |
| Second-line chemotherapy regimen | Dosing schemes |
| Taxane | 135 mg/m² every 3 weeks, up to 6 cycles |

a: carboplatin (area under the curve 5 mg/m²) or cisplatin (100 mg/m²) and 5-fluorouracil (1000 mg/m² per day for 4 consecutive days) every 3 weeks for six cycles. b: carboplatin (area under the curve 5 mg/m²) or cisplatin (75 mg/m²) and 5-fluorouracil (750 mg/m² per day for 5 consecutive days) every 3 weeks for six cycles.

**Table S4** Model parameters and baseline values for subgroup analyses.

| Subgroup | Sex | | Age category | |
| --- | --- | --- | --- | --- |
|  | Male | Female | < 65 years | ≥ 65 years |
| HR of OS (finotonlimab-chem vs. chemotherapy) | 0.78 | 0.47 | 0.69 | 0.83 |
| HR of PFS (cetuximab-chemo vs. chemotherapy) | 0.70 | 0.20 | 0.60 | 0.60 |
| HR of OS (pembrolizumab vs. cetuximab-chemo) | - | - | 0.82 | 0.79 |
| HR of OS (pem-chemo vs. cetuximab-chemo) | - | - | 0.83 | 0.52 |
| Subgroup | ECOG PS | | Disease status | |
|  | 0 | 1 | Metastatic | Recurrent only |
| HR of OS (finotonlimab-chem vs. chemotherapy) | 0.80 | 0.56 | 0.76 | 0.73 |
| HR of PFS (cetuximab-chemo vs. chemotherapy) | 1.80 | 0.40 | 0.60 | 0.60 |
| HR of OS (pembrolizumab vs. cetuximab-chemo) | 0.77 | 0.83 | 0.73 | 1.05 |
| HR of OS (pem-chemo vs. cetuximab-chemo) | 0.69 | 0.72 | 0.66 | 0.84 |

**Table S5** Results for subgroup analyses.

| **Group** | **Total cost ($)** | **LYs** | **QALYs** | **ICER vs. cetuximab-chemo** **($/QALY)** | **NMB ($)** | **INMB vs. cetuximab-chemo ($)** |
| --- | --- | --- | --- | --- | --- | --- |
| Sex male | | | | | | |
| Finotonlimab-chemo group | 31,958.74 | 2.23 | 1.70 | 2,061.05 | -11,495.81 | -29,983.94 |
| Cetuximab-chemo group | 39,397.03 | 1.32 | 1.01 | 1,211,379.67 | -27,940.76 | -46,428.89 |
| Sex female | | | | | | |
| Finotonlimab-chemo group | 64,604.18 | 1.56 | 1.17 | 203,689.23 | -7,113.39 | -25,601.52 |
| Cetuximab-chemo group | 93,045.75 | 1.32 | 1.08 | 808,571.53 | -84,979.89 | -103,468.02 |
| Age category < 65 years | | | | | | |
| Finotonlimab-chemo group | 32,638.09 | 2.42 | 1.84 | 2,527.77 | -10,397.07 | -28,885.20 |
| Cetuximab-chemo group | 44,421.42 | 1.32 | 1.02 | 802,220.76 | -33,183.64 | -51,671.77 |
| Pembrolizumab group | 51,601.10 | 1.92 | 1.46 | 46,093.04 | -34,126.10 | -52,614.23 |
| Pembrolizumab-chemo group | 66,292.05 | 1.82 | 1.35 | 102,988.12 | -51,361.14 | -69,849.27 |
| Age category ≥ 65 years | | | | | | |
| Finotonlimab-chemo group | 31,613.62 | 2.13 | 1.63 | 1,740.88 | -12,090.24 | -30,578.37 |
| Cetuximab-chemo group | 44,421.42 | 1.32 | 1.02 | 802,220.76 | -33,190.67 | -51,678.80 |
| Pembrolizumab group | 51,841.09 | 1.98 | 1.50 | 42,868.34 | -33,748.27 | -52,236.40 |
| Pembrolizumab-chemo group | 70,801.35 | 2.51 | 1.84 | 48,105.27 | -48,799.54 | -67,287.67 |
| ECOG PS 0 | | | | | | |
| Finotonlimab-chemo group | 31,818.07 | 2.19 | 1.67 | 1,942.91 | -12,110.14 | -30,598.27 |
| Cetuximab-chemo group | 22,456.58 | 1.32 | 0.99 | 636,376.32 | -10,275.47 | -28,763.60 |
| Pembrolizumab group | 52,006.78 | 2.02 | 1.53 | 40,743.74 | -33,845.40 | -52,333.53 |
| Pembrolizumab-chemo group | 68,051.24 | 2.08 | 1.54 | 69,845.25 | -49,846.92 | -68,335.05 |
| ECOG PS 1 | | | | | | |
| Finotonlimab-chemo group | 33,770.18 | 2.74 | 2.08 | 3,015.49 | -8,622.13 | -27,110.26 |
| Cetuximab-chemo group | 60,709.20 | 1.32 | 1.04 | 808,740.61 | -50,152.51 | -68,640.64 |
| Pembrolizumab group | 51,523.31 | 1.90 | 1.45 | 46,949.56 | -34,611.84 | -53,099.97 |
| Pembrolizumab-chemo group | 67,640.83 | 2.02 | 1.50 | 74,637.67 | -50,069.81 | -68,557.94 |
| Disease status Metastatic | | | | | | |
| Finotonlimab-chemo group | 32,103.03 | 2.27 | 1.73 | 2,174.42 | -11,009.44 | -29,497.57 |
| Cetuximab-chemo group | 27,603.44 | 0.87 | 0.67 | 8,771.60 | -19,688.22 | -38,176.35 |
| Pembrolizumab group | 52,352.53 | 2.10 | 1.59 | 37,170.13 | -32,710.69 | -51,198.82 |
| Pembrolizumab-chemo group | 68,482.04 | 2.15 | 1.59 | 64,632.71 | -50,616.79 | -69,104.92 |
| Disease status Recurrent only | | | | | | |
| Finotonlimab-chemo group | 32,326.47 | 2.33 | 1.78 | 2,322.00 | -10,906.42 | -29,394.55 |
| Cetuximab-chemo group | 44,421.42 | 1.32 | 1.02 | 802,220.76 | -33,077.78 | -51,565.91 |
| Pembrolizumab group | 50,056.10 | 1.54 | 1.18 | 110,161.50 | -36,833.28 | -55,321.41 |
| Pembrolizumab-chemo group | 66,180.45 | 1.80 | 1.34 | 105,710.35 | -51,020.24 | -69,508.37 |
